# Analysis of Transcription Factor Network Underlying 3T3-L1 Adipocyte Differentiation

**DOI:** 10.1371/journal.pone.0100177

**Published:** 2014-07-30

**Authors:** KyungOh Choi, Bassel Ghaddar, Colby Moya, Hai Shi, Gautham V. Sridharan, Kyongbum Lee, Arul Jayaraman

**Affiliations:** 1 Department of Chemical Engineering, Texas A&M University, College Station, Texas, United States of America; 2 Department of Chemical and Biological Engineering, Tufts University, Medford, Massachusetts, United States of America; Michigan State University, United States of America

## Abstract

Lipid accumulation in adipocytes reflects a balance between enzymatic pathways leading to the formation and breakdown of esterified lipids, primarily triglycerides. This balance is extremely important, as both high and low lipid levels in adipocytes can have deleterious consequences. The enzymes responsible for lipid synthesis and breakdown (lipogenesis and lipolysis, respectively) are regulated through the coordinated actions of several transcription factors (TFs). In this study, we examined the dynamics of several key transcription factors (TFs) - PPARγ, C/EBPβ, CREB, NFAT, FoxO1, and SREBP-1c - during adipogenic differentiation (week 1) and ensuing lipid accumulation. The activation profiles of these TFs at different times following induction of adipogenic differentiation were quantified using 3T3-L1 reporter cell lines constructed to secrete the *Gaussia* luciferase enzyme upon binding of a TF to its DNA binding element. The dynamics of the TFs was also modeled using a combination of logical gates and ordinary differential equations, where the logical gates were used to explore different combinations of activating inputs for PPARγ, C/EBPβ, and SREBP-1c. Comparisons of the experimental profiles and model simulations suggest that SREBP-1c could be independently activated by either insulin or PPARγ, whereas PPARγ activation required both C/EBPβ as well as a putative ligand. Parameter estimation and sensitivity analysis indicate that feedback activation of SREBP-1c by PPARγ is negligible in comparison to activation of SREBP-1c by insulin. On the other hand, the production of an activating ligand could quantitatively contribute to a sustained elevation in PPARγ activity.

## Introduction

With rising prevalence of obesity and related diseases, numerous studies have investigated the mechanisms underlying the expansion in body fat, i.e. white adipose tissue (WAT). The bulk of WAT cellular mass comprises metabolically active lipid-laden white adipocytes. *In vivo*, almost the entire adipocyte volume is filled by a single large lipid droplet, which expands or shrinks depending on the body's energy balance. Chronic overfeeding can lead to significant expansion of adipocyte volume, termed hypertrophy, to accommodate the storage of excess nutrients as lipids. In adult humans, the total number of mature adipocytes is thought to remain constant, as does the rate of turnover [1], [2]. Formation of new adipocytes through the differentiation of locally resident pool of precursor cells in the stromal fraction is essential for WAT remodeling [3]. However, net expansion of adipose tissue cellular mass is quantitatively driven by an increase in the size of mature adipocytes, as hyperplastic growth via recruitment of precursor cells increases the number of small, newly differentiated adipocytes that have a much smaller volume of fat stored [4].

At the cellular level, the fat content in adipocytes reflects a balance between enzymatic pathways leading to the formation and breakdown of esterified lipids, primarily triglycerides. This balance is extremely important, as both high and low lipid levels in adipocytes have deleterious consequences. Excessive accumulation of lipids, leading to cellular hypertrophy correlates strongly with accumulation of pro-inflammatory immune cells in WAT, which in turn underpins tissue insulin resistance and other metabolic alterations associated with obesity-related metabolic diseases [5]–[7]. Conversely, failure to sequester fatty acids in adipocytes due to inadequate lipid storage capacity is also not desirable, as this can lead to an elevation in circulating levels of free fatty acids and ectopic storage in other organs such as the liver [8].

The enzymes responsible for lipid synthesis and breakdown (lipogenesis and lipolysis, respectively) are regulated through the coordinated actions of several transcription factors (TFs) [9], [10]. *In vitro*, stimulation of growth-arrested preadipocytes with adipogenic hormones leads to an increase in the gene expression of CCAAT/enhancer binding proteins β (C/EBPβ) and δ (C/EBPδ) [11]. This is followed by an increase in the expression of two other TFs, peroxisome proliferator activated receptor-γ (PPARγ) and C/EBPα [12], which is thought to induce the enzymes that confer the recognizable metabolic phenotypes of the differentiated adipocyte [10], [13]. Along with sterol regulatory element binding protein-1c (SREBP-1c), PPARγ directly regulates the expression of several genes in lipogenesis [14], [15] as well as a key lipase that is transcriptionally regulated and specifically enriched in adipocytes (adipose triacylglycerol lipase, ATGL). Transcriptional regulation of lipogenesis also involves the two C/EBP isoforms β and α, which are sequentially activated during differentiation. A major target of these two isoforms is the gene encoding diacylglycerol acyltransferase 2 (DGAT2), which catalyzes the final esterification step in triglyceride synthesis.

The aforementioned TFs play a central role in regulating the adipocyte differentiation program, underscoring the complex interrelationship between molecular processes governing the formation and size expansion of adipocytes. In addition to C/EBP and PPARγ, several other TFs have been shown to impact adipogenesis. *In vitro* studies indicate that cyclic AMP response element binding protein (CREB) is another early transcriptional regulator of the adipogenic differentiation program that likely acts upstream of C/EBP. Increasing CREB activity through the addition of dibutyryl cAMP can induce differentiation in the absence of other conventional inducing agents, although this requires a very high (mM) concentration [9]. It has been shown SREBP-1c can enhance adipogenesis by increasing PPARγ expression [16]. Unlike CREB, however, SREBP-1c cannot directly initiate adipogenesis [14], and appears to depend on PPARγ for its own activation. Two other TFs, nuclear factor of activated T cells (NFAT) and forkhead transcription factor (FoxO1), appear to also modulate the activity of C/EBP and/or PPARγ. NFAT was demonstrated to form a composite enhancer complex with C/EBP and potentiate PPARγ expression [17], whereas FoxO1 has been shown to counter PPARγ activation in 3T3-L1 adipocytes [18].

These and other studies have led to significant progress in identifying the roles played by different TFs in regulating adipogenesis, and in some cases establishing activation/inhibition relationships between TFs. However, only limited data is available on the dynamics of the TFs in intact cells, particularly as the dynamics relate to the interaction between these regulatory molecules. In the context of obesity, achieving a desirable adipocyte phenotype, for example curbing lipid accumulation while maintaining differentiated adipocyte function, will likely require careful modulation of a regulatory network comprising several TFs whose dynamic activity profiles are interdependent. For example, the inhibition of PPARγ *in vivo* reduced adipogenesis, which is an expected outcome based on the known role of this TF in differentiation. However, this intervention also increased insulin resistance, one of the chief complications of type-2 diabetes mellitus [19].

Recently, Simicevic and coworkers reported on dynamic changes in absolute copy numbers of PPARγ and its co-receptor RXRα during differentiation of 3T3-L1 preadipocytes [20]. The authors formulated a quantitative model of PPARγ-DNA binding based on the copy number, local chromatin state and binding energetics. However, this study did not consider contributions from other TFs to the differentiation process. Another recent study investigated genome-wide changes of chromatin structure that accompany binding of TFs during differentiation of 3T3-L1 preadipocytes [21]. Using DNase I hypersensitive site analysis, this study found that multiple TFs (C/EBPβ, C/EBPδ, Stat5a, RXR, glucocorticoid receptor) cooperatively bind to the same site early in adipogenesis. The study also found that a subset of early remodeled C/EBPβ and C/EBPδ binding sites are occupied by PPARγ in the later stage of adipogenesis, suggesting a regulatory role of C/EBPβ and C/EBPδ for PPARγ binding. Taken together, these findings clearly indicate that temporal regulation of TF expression plays a critical role in adipocyte differentiation.

The goal of this study was to characterize the dynamics and interactions of the aforementioned TFs in adipocytes. To this end, we generated a separate (3T3-L1) cell line for each TF capable of reporting on the activation of the TF using secreted luciferase. We used these reporter cells to examine the TF dynamics both during adipogenic differentiation (week 1) and ensuing growth in adipocyte size due to lipid accumulation [22]. Our data show that TF activity generally peaks within one to two days following the induction of differentiation, and declines thereafter. The exception is the activity profile for PPARγ, which is characterized by an elevated plateau that is sustained well into the second week following adipogenic induction. To quantitatively analyze the observed profiles, we constructed a small interaction network involving a subset of the aforementioned TFs, namely CREB, C/EBPβ, PPARγ, and SREBP-1c, based on activation relationships reported in the published literature. The dynamics of this network was modeled using a combination of logical gates and ordinary differential equations. The model was trained and then evaluated against TF activity time profile data collected using the reporter cells. Model simulations and parameter sensitivity analysis suggest that the sustained elevation in PPARγ activity could reflect a positive feedback loop, where the PPARγ induces the production of an activating ligand (or ligands) via the induction of another TF, SREBP-1c.

## Results

### Generation of Gaussia luciferase reporter cell lines tracking transcription factor activity

**Figure 1:**
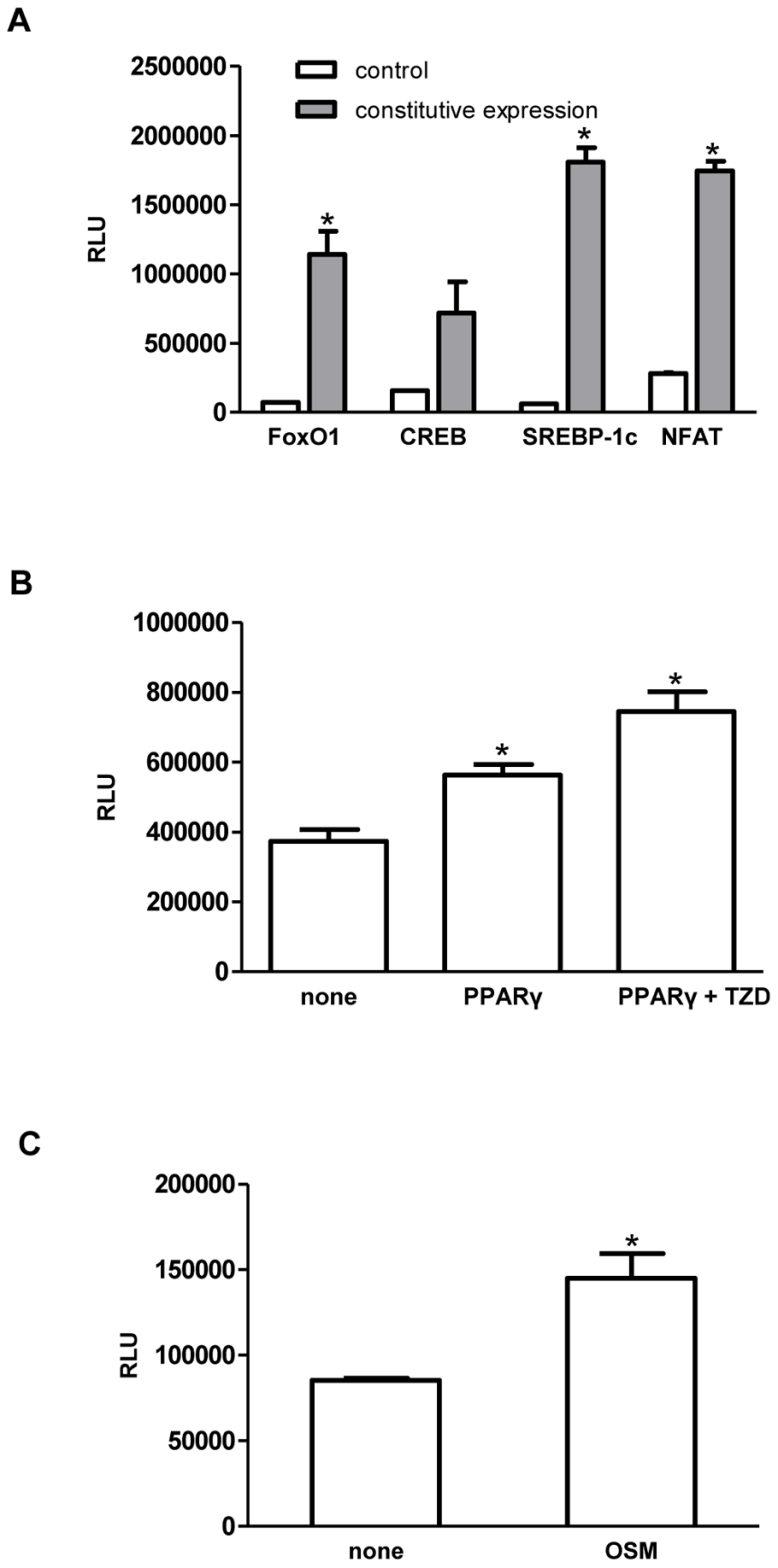
Validation of TF reporter constructs. (A) Foxo1, CREB, SREBP-1c, and NFAT3 reporter plasmids were validated by co-transfecting plasmids for constitutive expression of each TFs (pCMV5-FLAG-FoxO1, pCMV-Sport6-CREB, pSV Sport SREBP-1c, pEGFP-C1 NFAT3) and corresponding reporter plasmids into 293T/17 cells, and monitoring the TF-mediated Gaussia luciferase (Gluc) activity. A yellow fluorescence protein expressing plasmid (pEYFP-N1) plasmid was co-transfected with each reporter plasmid as a control. (B) The PPARγ reporter construct was verified by transfecting a plasmid containing the PPARγ gene was into 3T3-L1 PPARγ reporter cells, and activating PPARγ with 25 µM of rosiglitazone (RGZ). (C) The C/EBPβ reporter construct was validated by activating C/EBPβ in 3T3-L1 preadipocyte C/EBPβ reporter cells with the cytokine oncostatin M (OSM). Data represent mean ± SD. *: p<0.05.

A panel of six transcription factors (TFs) (PPARγ, SREBP-1c, NFAT, CREB, C/EBPβ and FoxO1) was chosen to develop a regulatory network underlying adipocyte differentiation and lipid loading. Reporter plasmids were developed as described in Materials and Methods and validated by determining if activation of a TF led to expression of the *Gaussia* luciferase gene from the reporter plasmid. For the TFs CREB, SREBP-1c, NFAT, and FoxO1, plasmids containing the full-length gene for each TF were over-expressed in 293T/17 cells by transfection along with the corresponding reporter plasmid. Figure 1A shows 5–30 fold increase in luciferase activity when the TFs were overexpressed for 48 h. The 3T3-L1 PPARγ reporter cell line was validated by transfecting the cells with a plasmid containing the PPARγ full length gene and activating the TF with 25 µM of a chemical agonist, rosiglitazone (RGZ). Figure 1B shows that overexpression of PPARγ increases PPARγ-driven luciferase activity by 1.3-fold, and stimulation with RGZ resulted in a 1.5-fold increase in luciferase activity. The C/EBPβ reporter was validated by stimulating 3T3-L1 preadipocyte C/EBPβ reporter cells with the cytokine oncostatin M (OSM), which resulted in a 1.7 fold increase in C/EBPβ-driven luciferase activity (Figure 1C). Together, these results confirmed the ability of the developed reporter cell lines to report activation of the different TFs.

**Figure 2:**
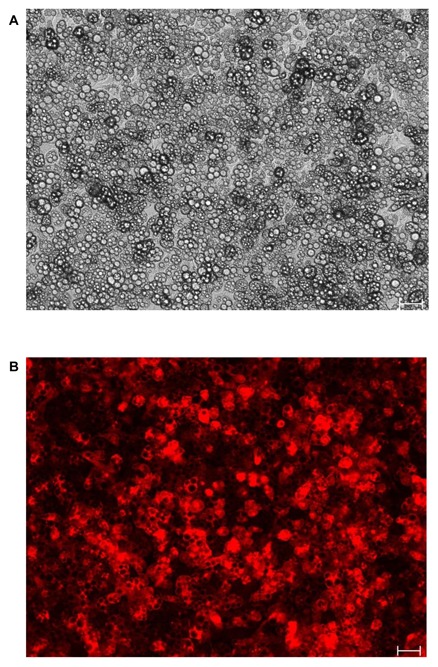
Representative images of PPARγ reporter cells 19 days post-induction. Micrographs show (A) transmitted light and (B) red fluorescence microscopy images. Scale bar  = 50 µm. Images for other TF (FoxO1, CREB, NFAT, SREBP-1c, C/EBPβ) reporter cell lines at the mature adipocyte state are available in Figure S1.

Lentivirus-mediated integration of the reporter plasmids into 3T3-L1 preadipocytes did not affect the ability of the different reporter cells to differentiate into adipocytes. Figures 2A & B show representative transmitted light and red fluorescence images of the PPARγ reporter cell line after 19 days in culture, and clearly demonstrate that adipocyte differentiation was not affected (i.e., reporter cells show comparable accumulation of lipid droplets and cell morphology to 3T3-L1 adipocytes without the reporter plasmid). Moreover, no differences in differentiation and lipid loading were observed between the different reporter cell lines (Figure S1). In addition, more than 90% of reporter cells demonstrated RFP expression, and suggest proper integration of the reporter DNA.

### Profiles of transcription factor activity during adipocyte differentiation and enlargement

**Figure 3:**
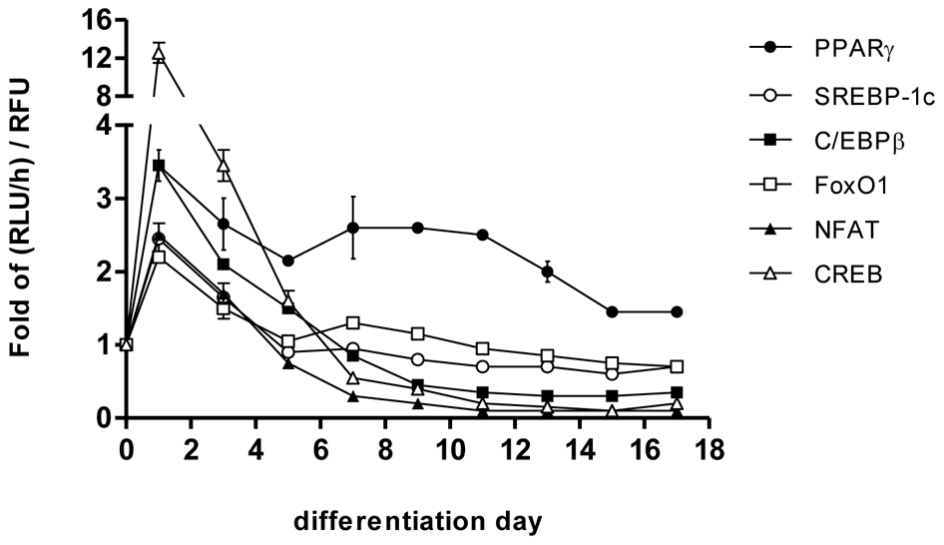
TF activity profiles. Gluc activity, which is a measure of TF binding activity, was measured every 24-medium change starting from day 0 when differentiation was induced to day 17. The rate of Gluc activity (RLU/h) was normalized with relative fluorescence units (RFU) measured at that time point. The fold change in the increase rate of Gluc activity was determined by normalizing the RLU/h/RFU value at each time point to the corresponding value at the start of differentiation (i.e., day 0). Data are from two independent experiments and represent mean ± SD.

3T3-L1 preadipocyte reporter cells were differentiated into adipocytes and the activity of each TF was monitored throughout the differentiation and maturation process. The different TFs demonstrated markedly different activation dynamics. As shown in Figure 3 and Dataset S1, CREB reporter cells demonstrated a pronounced 13-fold increase in normalized luciferase activity (RLU/h/RFU) on day1 after addition of differentiation media. However, this increase was transient as the normalized luciferase activity rapidly decreased and returned to day 0 levels by day 7. On the other hand, PPARγ reporter cells demonstrated a 3-fold increase in luciferase activity in the first 24 h after induction of differentiation. However, unlike CREB, this increase was sustained, as nearly 2-fold higher activation of PPARγ was observed until day 17 post-differentiation compared to that in preadipocytes. This trend was especially noticeable beyond day 7 post-differentiation. C/EBPβ, SREBP-1c, NFAT and FoxO1 all demonstrated similar activation dynamics with maximum activation observed at day 1 until day 3, and gradually decreasing to near-baseline values after day 5.

### Dynamic model of transcription factor interactions

**Figure 4:**
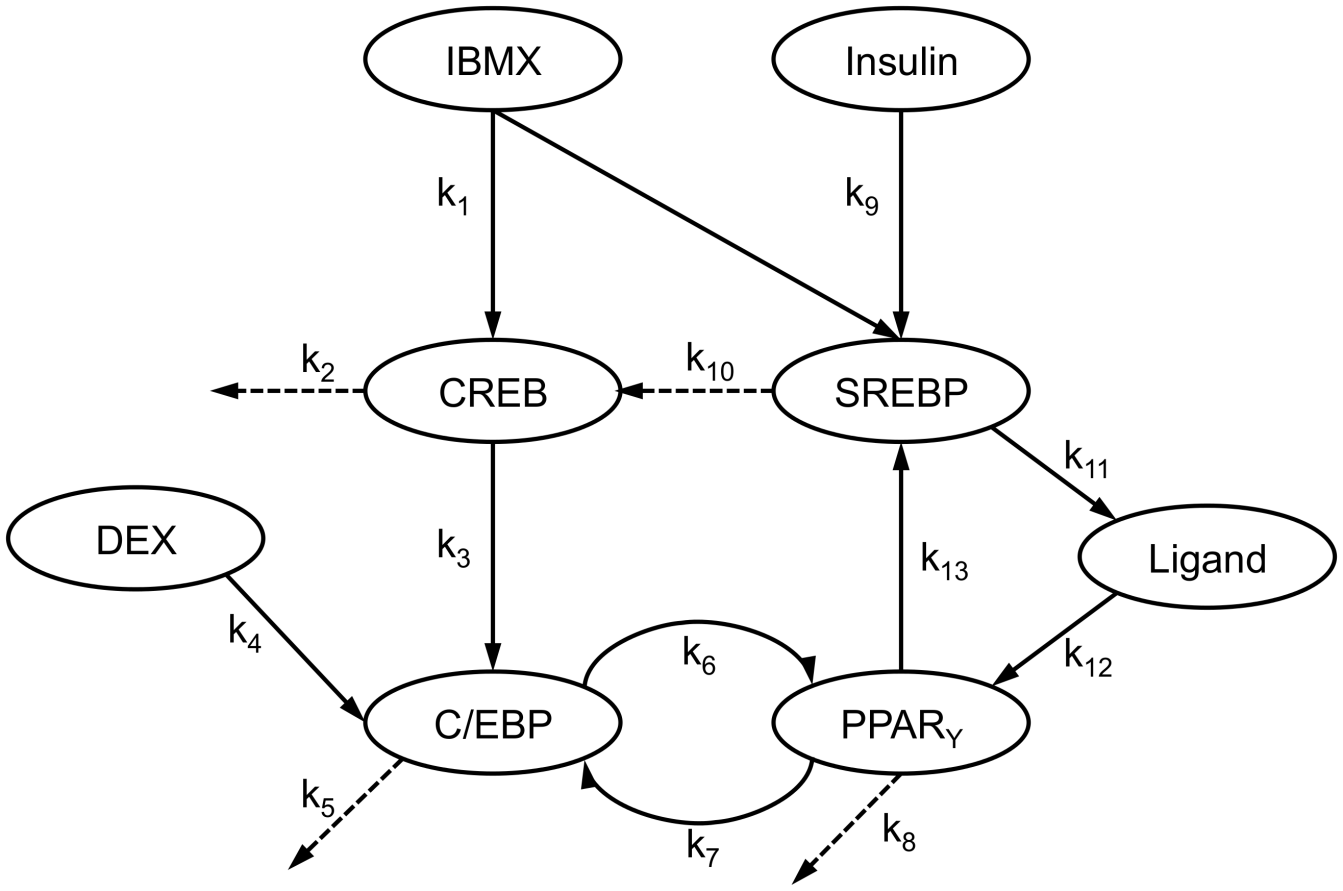
Schematic of TF network model. Arrows indicate direction of interaction. Model parameters labeling the dotted arrows (k_2_, k_5_, k_8_, and k_10_) represent first-order decay rate constants for the TFs. The rate constants shown in the schematic refer to the mass action models. See text for abbreviations.

Based on literature reports, a network model was assembled describing the interactions between TFs as well as their stimulation by adipogenic hormones (Figure 4). Only a subset of the measured TFs (CREB, C/EBPβ, PPARγ, and SREBP-1c) was considered, as the interactions involving NFAT and FoxO1 were less clearly documented in the published literature. Activation and decay of TFs were modeled using ordinary differential equations (ODEs) based on mass action kinetics (Table S1) or Hill equations (Table S2). Different possibilities regarding co-activation of TFs by upstream factors were modeled using logical AND/OR gates (Table S3), resulting in 64 model variants (32 mass action and 32 Hill equation models) (Table S4). The models' parameters (i.e. kinetic constants) were fitted to the normalized activity profiles (Figure 3) by minimizing the sum of squared residuals (SSR) between the measured and model-calculated TF activity profiles.

**Figure 5:**
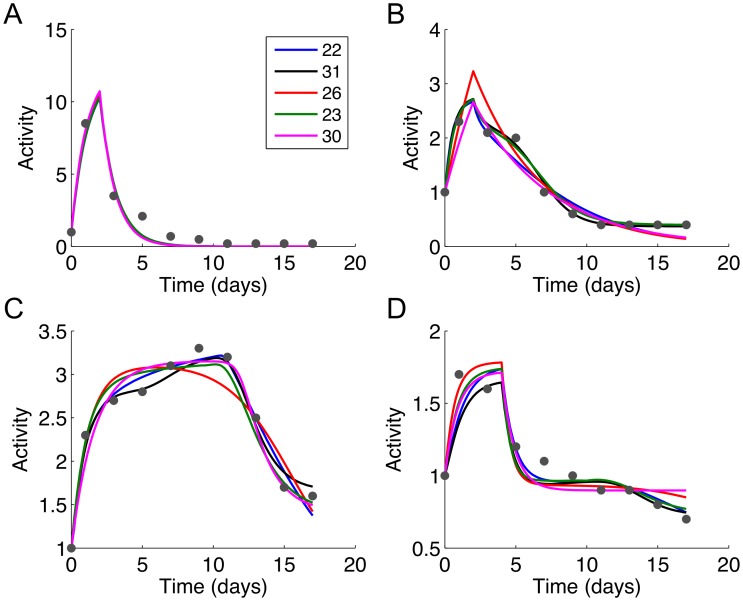
Simulated activity profiles for (A) CREB, (B) C/EBP, (C) PPARγ, and (D) SREBP-1c generated using the top five Hill equation models. The model numbers are shown in the figure legend, listed in order of increasing sum of squared residuals (SSR). The measured data (normalized mean Gluc activities, RFL/h/RLU) are shown as red dots. The specific combination of logic gates for these models can be determined from Tables S3 and S4.

**Figure 6:**
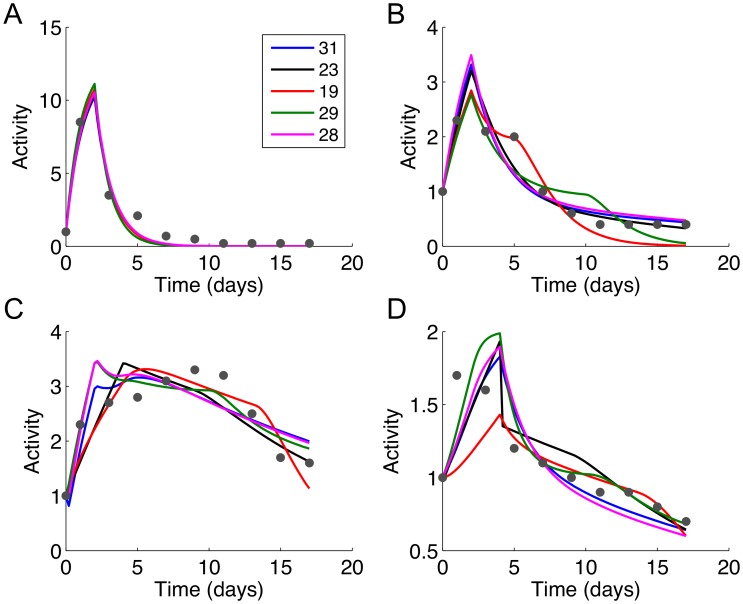
Simulated activity profiles for (A) CREB, (B) C/EBP, (C) PPARγ, and (D) SREBP-1c generated using the top five mass action models. The model numbers are shown in the figure legend, listed in order of increasing sum of squared residuals (SSR). The measured data (normalized mean Gluc activities, RFL/h/RLU) are shown as red dots. The specific combination of logic gates for these models can be determined from Tables S3 and S4.

The Hill equation models generally resulted in smaller SSR compared to the mass action models. The SSR for the mass action models ranged from 4.15 to 13.13 with a median of 7.47, whereas the SSR for the Hill equation models ranged from 3.05 to 5.47 with a median of 3.72 (Figure S2). Visual inspection of the simulated profiles corroborated the trends indicated by the SSR values. The TF activity profiles generated by the best fitting Hill equation models (Figure 5) showed smoother curves and more closely approximated the experimental data compared to the mass action models (Figure 6). Regardless of rank (based on SSR), The Hill equation models calculated essentially identical profiles for the TFs. In comparison, there were more noticeable differences between profiles calculated by the mass action models.

While the SSR values and calculated profiles appeared to suggest that the Hill equations provided the better model structure, the adjusted R^2^ values of these models were noticeably smaller (by 16.9%) than the corresponding R^2^ values (Table 1). The relatively large discrepancy in the two statistics indicated that the Hill equation models over-fit the available data. In comparison, the adjusted R^2^ values for the best fitting (top 8) mass action models were only slightly less (by 2.7%) than the corresponding R^2^ values. Eight of the 32 mass action models had adjusted R^2^ values greater than 0.90. Therefore, further analysis to discriminate between different model variants (AND/OR gate combinations) focused on these top 8 mass action models.

**Table 1:** Sum of squared residuals (SSR) and adjusted R^2^ values for the best ten mass action and Hill equation models representing different logical gate combinations.

	Mass Action				Hill Equation			
Rank	Model	SSR	R^2^	Adj. R^2^	Model	SSR	R^2^	Adj. R^2^
**1**	31	4.156	0.943	0.918	22	3.047	0.958	0.820
**2**	23	4.253	0.942	0.916	31	3.145	0.957	0.814
**3**	19	4.579	0.938	0.910	26	3.318	0.955	0.804
**4**	29	4.650	0.937	0.908	23	3.346	0.954	0.802
**5**	28	4.689	0.936	0.908	30	3.352	0.954	0.802
**6**	27	4.855	0.934	0.904	29	3.352	0.954	0.802
**7**	21	4.871	0.934	0.904	27	3.387	0.954	0.800
**8**	20	4.973	0.932	0.902	7	3.387	0.954	0.800
**9**	17	5.606	0.924	0.890	32	3.419	0.953	0.798
**10**	22	5.784	0.921	0.886	6	3.540	0.952	0.791

Every top mass action model (Table 1) included an OR gate at the SREBP-1c node (Table S3), indicating that insulin or PPARγ could independently activate this TF. The trend was less clear for the PPARγ node. At this node, half of the top 8 models combined the inputs C/EBPβ and ligand using an AND gate, whereas the other half used an OR gate. At the C/EBPβ node, 6 out of the top 8 models used an OR gate for the DEX input, including the best fitting model (MA31). The gate combination of the remaining two models, (CREB AND DEX) OR PPARγ, also suggested that DEX may not be a required factor for C/EBPβ activation, provided PPARγ is active. Indeed, PPARγ alone was sufficient to activate C/EBPβ in 4 out of the 8 models, with the other 4 models requiring both CREB and PPARγ in the absence of DEX. While PPARγ appeared to play an essential role in C/EBPβ activation, the role of CREB was more ambiguous. This ambiguity may reflect the limited specificity of the reporter construct used in this study, which could not differentiate between different C/EBP isoforms. As the reporter could respond to activation of C/EBPα, -β, or -δ, it is possible (even likely) that the TF data used to train the models represent the aggregate responses of all three isoforms, whereas only the -β isoform depends on CREB [23].

### Evaluation of model fit

Qualitatively, the TF time profiles generated using the best fitting mass action model (MA31) were largely consistent with the measured data. The calculated profiles correctly ordered the peak activities for the TFs, and showed a sustained elevation in PPARγ activity extending to day 11 post-induction. On the other hand, there were differences in the shapes of the calculated profiles compared to the trends implied by the measured data. To characterize the differences, we utilized a bootstrapping method to estimate the confidence intervals (CIs) for the parameters and variables of the model.

**Figure 7:**
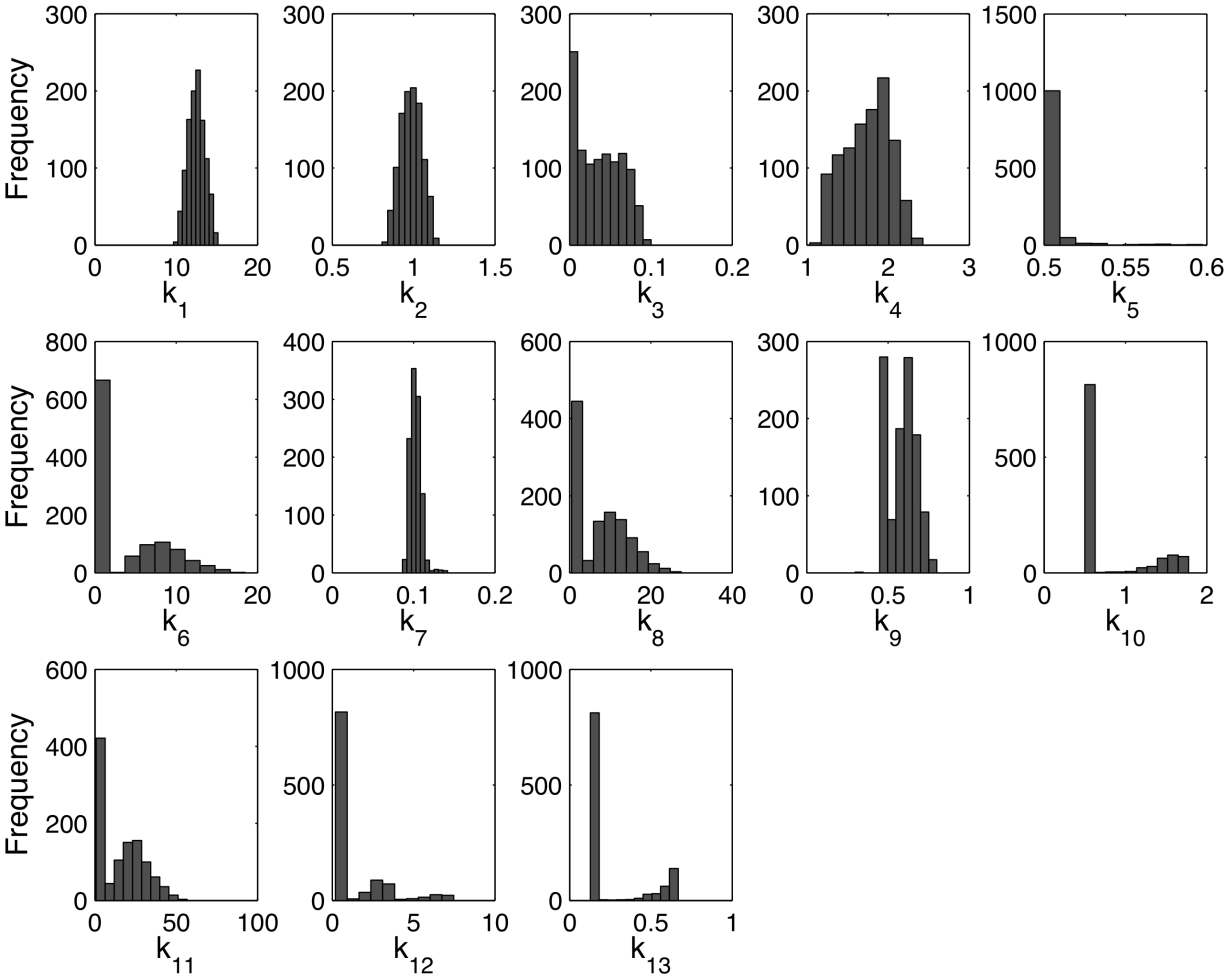
Parameter distributions for the best fitting mass action model (MA31). Note that the x-axis scales have been adjusted to show the shapes of the distributions. The distributions were obtained using a bootstrapping method by twice repeating the parameter optimization 2,000 times on noise-added data (see Materials and Methods, Parameter estimation).

From the parameter distributions (Figure 7) and CI shape values (L) (Table S1), it can be seen that the model was nonlinear with respect to the parameters, especially *k_5_, k_10_, k*_12_, and *k_13_*, which had shape values 8.62, 3.72, 5.78, and 3.31, respectively. The CI lengths suggested that most of the parameters were well constrained. For 9 out of the 13 kinetic parameters, the CI lengths were of the same order magnitude or smaller than the median estimated parameter value (Table S1), ranging from 10^−2^ to 10^0^. The most tightly constrained parameters (L∼10^−2^) related to C/EBPβ: *k_3_* (activation of C/EBPβ by CREB), *k_7_* (activation of C/EBPβ by PPARγ) and *k_5_* (degradation constant for C/EBPβ). Larger CI lengths (on the order of 10^1^), and thus greater uncertainty, were calculated for the parameters characterizing the activation of PPARγ by C/EBPβ (*k_6_*), induction of ligand production by SREBP (*k_11_*), and degradation of PPARγ (*k_8_*).

**Figure 8:**
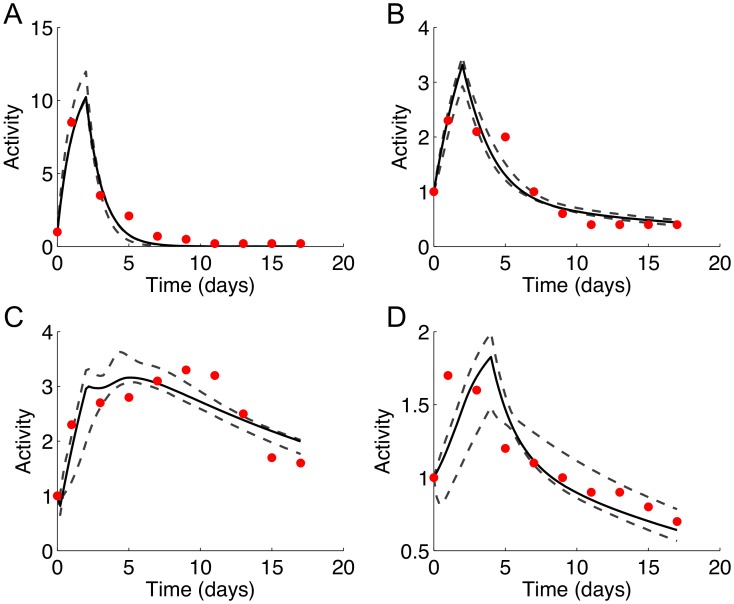
Simulated activity profiles for (A) CREB, (B) C/EBP, (C) PPARγ, and (D) SREBP-1c generated using the best fitting mass action model (MA31). Dashed lines show 95% confidence intervals. The confidence intervals at each time point were calculated based on the percentiles obtained from repeated simulations using the bootstrap method (see Materials and Methods, Sensitivity analysis for details). The measured data (normalized mean Gluc activities, RFL/h/RLU) are shown as red dots.

To investigate the consequences of parameter uncertainty on the simulated profiles, 95% CIs were computed for the TFs and ligand from their distributions obtained using the bootstrap parameters. Approximately 25% of the measured data points were outside the calculated CIs. Specific discrepancies between simulated and experimental profiles could be noted by locating the data points lying outside the confidence intervals (Figure 8). For example, the simulated profile for C/EBPβ (Figure 8B) showed an initially sharp rise until day 2, followed by a rapid decline, whereas the measured profile showed a broader peak extending from day 1 to day 5. For SREBP-1c (Figure 8D), the simulated profile showed a delay in the peak activity relative to the measured data. Overall, the shape differences in the TF profiles reflected a sharper rise or fall of the simulations relative to the measured data, presumably due to the simplifying assumptions regarding the concentrations of the hormones (DEX, IBMX, and insulin), which were modeled as step inputs.

### Parameter sensitivity

To assess the sensitivity of the model simulations to the estimated parameter values, a series of numerical perturbation experiments were performed. Each perturbation experiment decreased/increased the value of a single parameter by a fixed fraction (e.g. 15%) while keeping all other parameters constant, and calculated the change in TF and ligand time profiles. Similar to the CI calculations, the perturbation experiments used a sampling based approach, resulting in a distribution of changes for each model species. Statistically, one-sample *t*-tests performed on the changes showed that every parameter had a significant (non-zero) impact (p<0.05) on at least one model species when the parameter was decreased (or increased) by 15%, with most parameters significantly affecting multiple model species (Dataset S2). To obtain a more detailed analysis, we calculated an average perturbed time profile for each TF or ligand, and compared this perturbed profile against the 95% CIs of the corresponding unperturbed profile. Table 2 summarizes the results of this analysis for the case when each parameter was decreased or increased by 15% from its baseline (unperturbed) value. Based on the fraction of perturbed data points that lie outside the CIs, the ligand profile was the least sensitive across all parameters, presumably due to the large uncertainly of this profile, which was not experimentally measured in this study. The CREB profile was also largely insensitive to small perturbations, with the exception of values assigned to *k_1_* and *k_2_*, which characterize CREB activation by IBMX and turnover of the TF, respectively. The other three model species all exhibited large sensitivities, especially with respect to the degradation constant of PPARγ (*k_8_*), degradation constant of SREBP-1c (*k_10_*) and rate constant for ligand synthesis (*k_11_*). Similar trends were obtained for larger perturbations (Dataset S3).

**Table 2:** Fraction of model calculated data points that lie outside of the 95% confidence interval of the baseline case after a parameter has been perturbed.

Perturbed Parameter	CREB		C/EBP		PPAR		SREBP		Ligand	
	-	+	-	+	-	+	-	+	-	+
**1**	0.216	0.216	0.011	0.011	0.011	0.011	0.011	0.011	0.011	0.011
**2**	0.921	0.796	0.011	0.011	0.011	0.011	0.011	0.011	0.011	0.011
**3**	0.011	0.011	0.011	0.011	0.011	0.011	0.011	0.011	0.011	0.011
**4**	0.011	0.011	0.148	0.136	0.011	0.011	0.011	0.011	0.011	0.011
**5**	0.011	0.011	0.875	0.875	0.682	0.477	0.011	0.011	0.011	0.011
**6**	0.011	0.011	0.080	0.102	0.602	0.557	0.011	0.011	0.011	0.011
**7**	0.011	0.011	0.648	0.636	0.011	0.011	0.011	0.011	0.011	0.011
**8**	0.011	0.011	0.693	0.671	0.864	0.852	0.784	0.716	0.011	0.011
**9**	0.011	0.011	0.136	0.171	0.693	0.682	0.182	0.102	0.011	0.011
**10**	0.011	0.011	0.648	0.625	0.830	0.807	0.875	0.750	0.011	0.011
**11**	0.011	0.011	0.648	0.659	0.830	0.841	0.716	0.750	0.011	0.011
**12**	0.011	0.011	0.034	0.011	0.057	0.011	0.011	0.011	0.011	0.011
**13**	0.011	0.011	0.602	0.614	0.784	0.784	0.727	0.784	0.011	0.011

Plus and minus columns refer to a 15% increase and decrease in the parameter value relative to the baseline case, respectively.

### Perturbation of the transcription factor network by forskolin treatment

**Figure 9:**
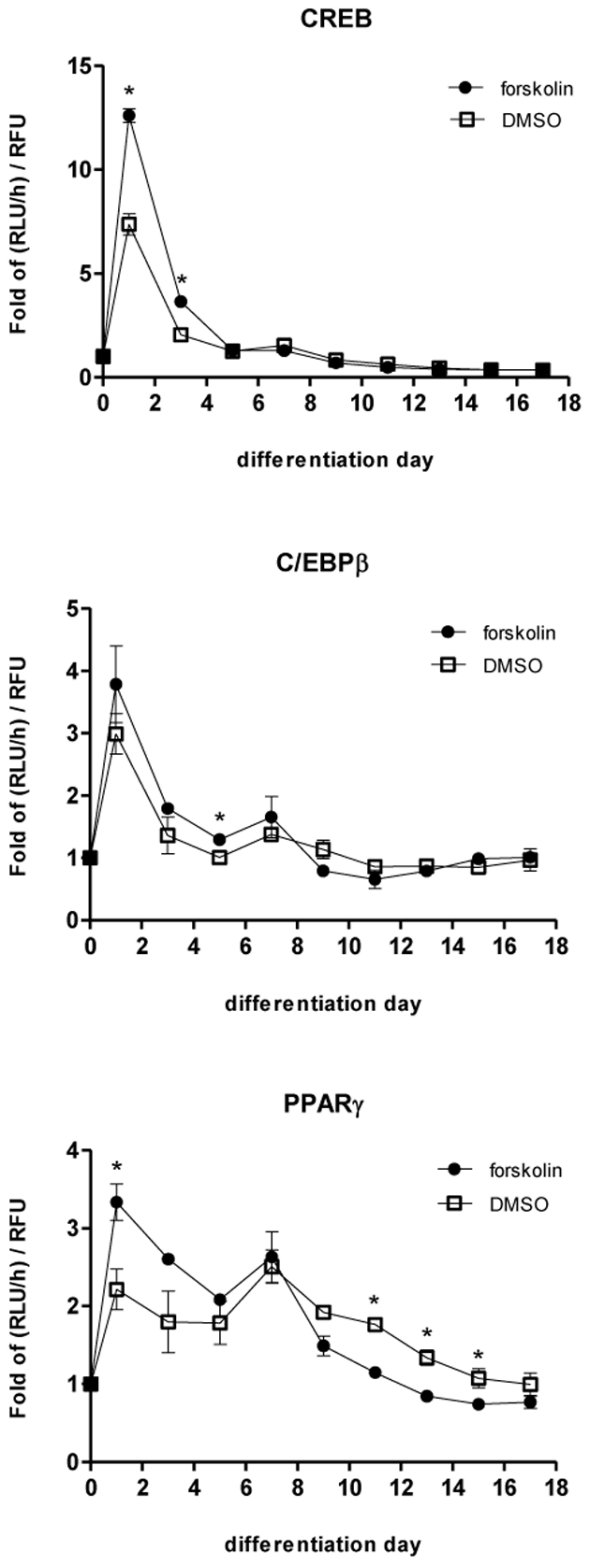
Perturbation of CREB activity profiles. (A) Perturbation of CREB activity profiles upon addition of 10 µM of forskolin or 0.1% DMSO at day 0 for 48 h. (B) and (C) show activation of C/EBPβ and PPARγ, respectively, with forskolin treatment as compared to DMSO control. The RLU/h/RFU for each time point was normalized to the value at the start of differentiation (day 0). Data are from two independent experiments and represent mean ± SD. *: p<0.05.

**Figure 10:**
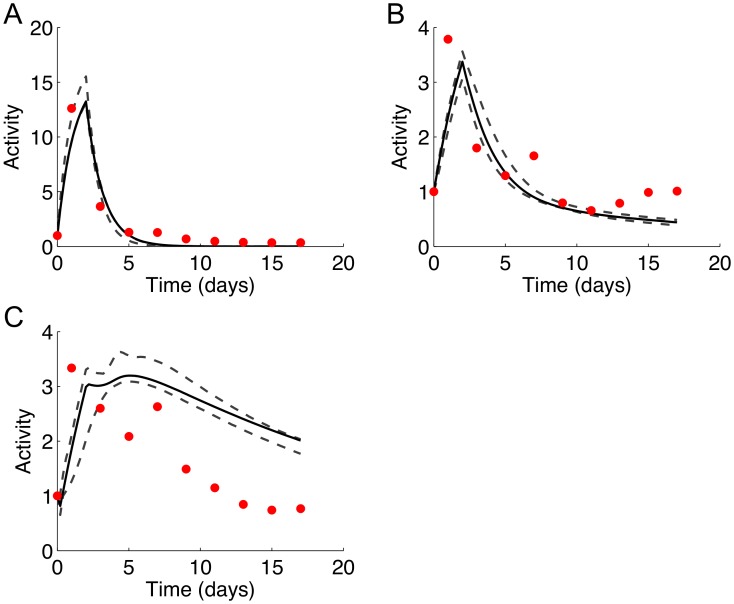
Simulated activity profiles for (A) CREB, (B) C/EBP, and (C) PPARγ generated using the best fitting mass action model with added forskolin input. Forskolin treatment was modeled as a step increase in IBMX during the first induction period (days 0 to 2). All other model parameters (*k_1_*–*k_13_*) were kept at the same values that were estimated from the training data without forskolin. Dashed lines show 95% confidence intervals. The measured data (normalized mean Gluc activities, RFL/h/RLU) are shown as red dots.

In order to evaluate the model with a new dataset, we experimentally perturbed the TF network using forskolin, and investigated its effects on the dynamics of C/EBPβ and PPARγ. Similar to IBMX, forskolin raises the intracellular concentration of cAMP, which in turn activates CREB. Therefore, the addition of forskolin was modeled as an increase in the concentration of IBMX relative to the unperturbed condition. As shown in Figure 9A and Dataset S4, forskolin treatment on day 0 resulted in 1.7-fold increase in the maximum activation of CREB as compared to the control, while retaining the same activation dynamics. Interestingly, the activation of C/EBPβ (Figure 9B and Dataset S4) and PPARγ (Figure 9C and Dataset S4) were more muted, increasing only 1.3-fold and 1.5-fold, respectively, compared to the control condition receiving only the adipogenic induction cocktail. These results are qualitatively consistent with the model simulations, which also predicted an attenuation of forskolin's effect downstream of CREB (Figure 10). In the simulations shown in Figure 10, the IBMX level was set to 1.4-fold above the control condition, which resulted in the optimal fit between the simulated profiles and experimental data. Adjusting the IBMX level primarily affected the peak activity of CREB, and had little effect on the C/EBPβ and PPARγ profiles (Figure S3). This observation is consistent with the results of the parameter sensitivity analysis, which showed that the rate constant for activation of C/EBPβ by CREB (k_3_) had little effect on any of the model species.

## Discussion

In this study, we assembled an interaction network model of several key TFs whose concerted actions are suspected to regulate adipocyte differentiation and lipid storage. We trained this model using activation time profiles of the TFs measured over a nearly three-week long period after inducing the differentiation of 3T3-L1 preadipocytes. The activity profiles were obtained using reporter cells generated from 3T3-L1 cells. In these reporter cell lines, binding of a TF to its recognition sequence leads to expression of a *Gaussia* luciferase reporter gene. Since the luciferase gene has a secretion leader sequence, non-invasive dynamic profiling of TF activity is possible by monitoring the culture supernatant for luciferase activity. This approach enabled us to directly monitor TF function (i.e., binding to its recognition sequence) as opposed to inferring activity from mRNA or protein data. The non-invasive measurement technique also allows for frequent sampling, which, in turn, facilitates collection of data for model construction and testing. Lastly, this approach also allows for TF activation profiles to be measured from the same batch of cells in a single experiment from differentiation to maturation.

The differentiation of preadipocytes into adipocytes is characterized by the appearance of microscopically visible intracellular lipid droplets and rounding of the cell shape, and is mediated by multiple enzymes and regulatory TFs. While previous data on the activation of different TFs underlying adipocyte differentiation and enlargement are available in the literature, differences in the experimental conditions and readout methods limit their utility for developing an integrated network model. In this study, we address this by developing a TF model using time-course data from the same cell population.

Our results show that different TFs demonstrate distinct activation profiles during adipocyte differentiation and enlargement, which are consistent with published reports. The temporal correlation between CREB and C/EBPβ is one such example (Figure 3) where activation of C/EBPβ follows the activation of CREB until day 5. Zhang and coworkers [23] showed that CREB induces expression of C/EBPβ at 8 h after induction of the adipogenic program. Similarly, SREBP-1c has been shown to promote adipogenesis by inducing the enzymatic production of a PPARγ ligand [14]. It has also been suggested that SREBP-1c could transcriptionally activate PPARγ by directly binding to a PPARγ promoter [16]. Another TF implicated in the regulation of PPARγ is NFAT, based on the evidence that NFAT associates physically with PPARγ [24]. Our data show that both SREBP-1c and NFAT demonstrate activation profiles comparable to C/EBPβ, which is known to precede and activate expression of PPARγ [25], during the initial differentiation phase (days 1-3). Thus, our data provide corroborating evidence for SREBP-1c and NFAT in regulating PPARγ activity.

Activation of PPARγ is critical for terminal differentiation and maintenance of differentiated phenotype. Our data also show that PPARγ activity increased gradually from the induction of differentiation and stayed elevated between days 7 and 11. While the activation levels decreased after day 11, they were still significantly higher than pre-induction levels for the duration of the experiment. Interestingly, only PPARγ demonstrated sustained activation as the activity of other TFs decreased to pre-induction levels after adipocyte maturation (days 7–10). This result is consistent with the primary role for PPARγ in adipocyte differentiation and enlargement. Interestingly, FoxO1 did not show significant activation over the time course of the experiment. A recent study has shown that a direct protein-protein interaction leads to repression of PPARγ activity by FoxO1 [26]. However, since FoxO1 interferes with PPARγ by recruitment to the PPARγ response element, measurement of FoxO1 binding to its own response element may not show any correlation to alterations in PPARγ activity.

To more quantitatively characterize the dynamics of the TFs, we assembled a mathematical model based on interactions documented in the published literature. For four of the TFs monitored in this study, namely CREB, C/EBPβ, PPARγ, and SREBP-1c, we found a general consensus regarding the activation cascade, with CREB and SREBP-1c upstream of C/EBPβ and PPARγ, respectively, and positive feedback between C/EBPβ and PPARγ. For the sake of parsimony, we modeled these interactions as a sequence of direct activation events between TFs. The exception was the activation of PPARγ by SREBP-1c, as there was strong evidence that SREBP-1c activation induces the production of one or more endogenous metabolite ligands for PPARγ [27]. We also explored different possibilities with regards to co-activation requirements of a downstream TF for cases where the TF could be activated by more than one TF and/or hormonal input. We modeled these possibilities using a logic-based modeling approach, which has been utilized in various forms to describe the input-output dynamics of cellular signaling networks [28]–[30]. As the TF activities varied continuously, rather than discretely, we decided to utilize ODEs in conjunction with logical gates, similar to the recent work by Kraeutler et al. [31]. While other approaches such as fuzzy logic modeling could also be used to approximate continuous variations in the model species [30], this would require additional adjustable parameters [32] and/or manual refinement [33], thus undermining a major benefit of logic-based modeling, namely simplicity. Along this vein, we compared the fits achieved using Hill equations against simpler mass action kinetics. We found that the improvement in the SSR achieved using Hill equations essentially reflected a larger degree of freedom, as indicated by the adjusted R^2^ statistic (Table 1), which penalizes over-fitting. Consequently, the Hill equation models could not adequately discriminate between different logic gate combinations based on goodness of fit. Moreover, there was no mechanistic basis, e.g. known cooperative interactions between the TFs modeled in this study, to support the selection of a more complex functional form to describe the activation kinetics. For these reasons, we focused on the first-order mass action models in evaluating potential co-activation at various network nodes using different logical gate combinations.

We found that an all OR-gate model best explained the data collected in this study, although an alternative model with an AND gate at the PPARγ node fit the data nearly as well. This suggests that PPARγ activation by C/EBPβ may require a ligand co-activator, which in turn is produced by an enzyme induced by SREBP-1c. Due to its prominent role in regulating adipogenesis and differentiated adipocyte functions, ligand activation of PPARγ has been extensively studied using high-affinity synthetic agonists, notably TZDs, and more recently, certain environmental chemicals. As a nuclear receptor, PPARγ is capable of recognizing a variety of oxidized fatty acids and related lipid metabolites, including prostaglandins [34]–[36]. Tzameli and coworkers constructed a beta-galactosidase-based PPARγ ligand-sensing vector system to show that one or more endogenous ligands are produced following the induction of adipogenesis in response to an elevation in cAMP [37], but establishing the chemical identity of these ligands has been challenging. Metabolic products of the arachidonate cyclooxygenase (COX) pathway, particularly 15-deoxy-12,14 prostaglandin J_2_ (15-dPGJ_2_), have been shown to be potent activators capable of inducing adipogenesis [38]. However, it remains an open question as to whether differentiating adipocytes synthesize 15-dPGJ_2_ or other suspected natural lipid ligands at sufficiently high concentrations *in vivo* to activate 15-dPGJ_2_ [39]. In the present study, we did not assume a specific identity for the natural ligand, and thus could not experimentally monitor its concentration, which clearly contributed to the uncertainty regarding co-activation of PPARγ. In this regard, establishing the identity of the natural ligand and tracking its concentration will be crucial in further resolving the dynamics of the adipocyte TF network.

As published data on adipocyte TF network activation rates were not available, the rate parameters were estimated from the measured profiles collected in this study. Confidence interval calculations and parameter sensitivity analysis suggested that most of the parameters were well constrained, and that the model species were sensitive to the parameters, allowing the model fitting procedure to estimate the parameters to within one order of magnitude. Outside of the general assumption that the rate constants have to be nonnegative, the only other constraint applied to the parameters was to set a lower bound for the turnover rates of the TFs. Based on the estimated parameter distributions, the decay rate constants ranged from ∼0.5 day^−1^ for C/EBPβ (k_5_) to ∼7.2 day^−1^ for PPARγ (k_8_) (Table S1 and Figure 7). These decay rate constants correspond to half-life ranges of ∼33 to ∼2.3 h, which are comparable to published turnover rates of TFs [40]–[45].

Compared to the other rate constants, the decay rate constant for PPARγ had a relatively large degree of uncertainty, as did two other rate parameters for the activation or degradation of this TF. The simplest explanation is the lack of direct and specific experimental observations on the dynamics of the putative ligand. The PPARγ activity profile intimately depends on the ligand profile through *k_11_* (ligand synthesis rate constant), another parameter with a relatively large CI length, as shown by the sensitivity analysis (Table 2). It is likely that multiple PPARγ ligands are produced in differentiating adipocytes [46], although a dominant endogenous ligand has not yet been conclusively identified. Another, related limitation regarding the interpretation of the simulated ligand time profile is that it is not possible to determine an absolute concentration range for the ligand, because normalized TF time profiles were used in fitting the model.

Despite these limitations, the mathematical model revealed several noteworthy features of the adipocyte TF network. First, model simulations of forskolin stimulation showed that an increase in CREB activity does not propagate to C/EBP and PPARγ, indicating that the latter two TFs receive additional inputs (from DEX and ligand) having a quantitatively larger impact than the input from CREB. This simulation was in good agreement with measured data, which also showed little increase in TF activity downstream from CREB in response to forskolin treatment. Second, the estimated values for rate parameters suggest that feedback activation of SREBP-1c by PPARγ is negligible in comparison to activation of SREBP-1c by insulin, corroborating the downstream placement of SREBP-1c relative to PPARγ in recently published studies [47], [48]. Third, the estimated parameters suggest that the strength of the positive activation loop between C/EBP and PPARγ is comparable to the strengths of the activation cascades connecting the TFs to hormonal inputs. This finding is consistent with previous experiments showing that direct (e.g. genetic) manipulation of either C/EBP or PPARγ could drive the adipogensis program to similar extents as hormonal stimulation [49], [50].

Prospectively, the TF reporters and model described in this study could provide a set of useful tools for investigating methods to modulate adipocyte differentiation and metabolism by intervening at the level of the regulatory network. For example, we could simulate overall change of activity profiles for key TFs triggered by a specific perturbation (i.e. exposure to an agonist or antagonist) and its influence on expression levels of target genes such as adipocyte metabolic enzymes that are regulated by the TFs. We expect the systematic understanding of the TF network also to lead to the development of new treatment approaches against obesity, and for studying other phenotypes regulated by the coordinated activity of TFs.

## Materials and Methods

### Materials

3T3-L1 cells were kindly provided by Prof. Barbara Corkey (Boston University School of Medicine, MA). Tissue culture reagents including Dulbecco's Modified Eagle's Medium (DMEM), calf serum (CS), fetal bovine serum (FBS), human insulin, and penicillin/streptomycin were purchased from Invitrogen (Carlsbad, CA). Unless otherwise noted, all other chemicals were purchased from Sigma (St. Louis, MO).

### Cell culture and differentiation

3T3-L1 cells were seeded in 6-well tissue culture plates in preadipocyte growth medium consisting of DMEM supplemented with CS (10% v/v), penicillin (200 U/ml) and streptomycin (200 µg/ml). Medium was replenished every other day. Two days post-confluence, differentiation was induced using a standard adipogenic cocktail (1 µg/ml insulin, 0.5 mM isobutylmethylxanthine, and 1 µM dexamethasone) added to a basal medium (DMEM with 10% FBS and penicillin/streptomycin). After 48 h, the first differentiation medium was replaced with a second differentiation medium consisting of the basal adipocyte medium supplemented with only (1 µg/ml) insulin. After another 48 h, the second medium was replaced with the adipocyte basal medium and replenished every other day.

### Construction of *Gaussia* luciferase (Gluc) reporter plasmids

Lentiviral reporter plasmids for monitoring activation of six transcription factors (TFs) - PPARγ, SREBP-1c, NFAT, CREB, C/EBPβ and FoxO1 - during adipocyte differentiation and lipid loading were constructed as described below. For each TF, consensus binding sites (response elements; RE) in target gene promoter regions were identified using TRANSFAC database 7.0 Public (AGGACAAAGGTCA for PPARγ, CATGTG for SREBP-1c, GGAAAATTTGAGTCA for NFAT, TGACGTCA for CREB, ATTGCGCAAT for C/EBPβ and AGTTGGACGCGAC for FoxO1). Response element (RE) oligonucleotides containing the binding sequence for each TF were chemically synthesized. Each RE oligonucleotide consists of three consensus binding sequences separated by 4–6 bases (spacer sequence) and a unique restriction enzyme (*EcoR*I and *Afe*I) cleavage sites at the ends. The RE oligonucleotides were cloned into Gluc-DRE2-viral vector (pCS-sMAR8-pA1-DRE2-hPGK-cHS4-tACTB-SPA-Gluc-CMVmin) [51] in which expression of the *Gaussia* luciferase (Gluc) is under the control of a minimal promoter. Expression of Gluc is induced only when a TF binds to its consensus binding site. In addition, a plasmid containing a non-specific binding sequence (AGTTGGACGCGAC) was constructed to generate a control cell line that expresses a basal level of Gluc in a TF independent manner. Clones containing the correct RE were identified by multiple restriction enzyme digests and sequenced to verify fidelity.

### Generation of stable reporter cell lines

Stable reporter cell lines for each TF were generated by lentiviral transduction. To produce lentiviral particles, each TF reporter plasmid and two helper plasmids (psPAX; Addgene plasmid 12260 and pMD2.G; Addgene plasmid 12259, Dr. Trono, Lausanne, Switzerland) were co-transfected into 293T/17 cells using the calcium phosphate transfection method [52]. After 24 h following the transfection, the medium was replenished and 5 mM of sodium butyrate was added, and incubated for an additional 24 h. Supernatants containing viral particles were collected, pooled, filtered with 0.45 µm filters, and centrifuged for 2 h at 4°C at 48000 ×g. The viral titer was measured using a Lenti-X qRT-PCR titration kit (Clontech, Palo Alto, CA). To transduce 3T3-L1 preadipocytes, concentrated virus particles (∼2×10^8^ IFU) were added to the cells in presence of Polybrene (hexadimethrine bromide). The cells were incubated with the virus particles for 15 h, and the medium was replenished the next day. The efficiency of transduction was assessed by microscopic analysis.

### Validation of reporter plasmid function

Plasmids for constitutive expression of the TFs were purchased from Addgene (pCMV5-FLAG-FoxO1, pSV Sport SREBP-1c, pEGFP-C1 NFAT3, pcDNA flag PPAR gamma) or Invitrogen (pCMV-Sport6-CREB). 293T/17 cells were seeded in 6-well tissue culture plates, and ∼1 µg of each expression plasmid was cotransfected along with the corresponding reporter plasmid (∼1 µg) using the calcium phosphate transfection. For control experiments, the same amount of pEYFP-N1 plasmid (constitutive expression of yellow fluorescent protein) was transfected. At 48 h post-transfection, supernatants were collected and luciferase activity measured using the BioLux Gaussia Luciferase Flex assay kit (New England Biolabs, Ipswich, MA). Additionally, the PPARγ reporter plasmid was validated using thiazolidinedione (TZD) as the agonist. PPARγ was overexpressed from plasmid (pcDNA flag PPAR gamma) in 3T3-L1 PPARγ reporter cells and 25 µM TZD was used to activate PPARγ for 24 h. The C/EBPβ reporter cell line was validated by up-regulating C/EBPβ with 100 ng/mL oncostatin M (OSM) for 12 h [53]. Luciferase activity in the supernatant was determined as described above.

### Measurement of transcription factor activity profiles

For profiling TF activation in each reporter cell line, 3T3-L1 preadipocyte reporter cells for each TF were seeded in 6-well tissue culture plates and differentiated into adipocytes as described above. At different stages post-differentiation, 30 µL supernatant samples were collected at 24 h post-medium change from day 0 (induction of differentiation) until day 17. Samples were stored at −20°C prior to assessing luciferase activity. The luciferase activity (Relative Light Units; RLU) measured was used to calculate the rate of Gluc production (RLU divided by the time over which Gluc was secreted). As the red fluorescence intensity (Relative Fluorescence Units; RFU) measured at 550 nm (excitation) and 600 nm (emission) scales linearly with cell density (Figure S4A) and the Gluc activity (RLU) also correlates with RFU (Figure S4B), the rate of Gluc production was also normalized with the RFU to account for differences in cell density between different experiments. The fold-increase in TF activity was determined by normalizing the (RLU/h)/RFU at each time point to the corresponding value at the start of differentiation (i.e., day 0).

### Perturbation of the transcription factor network by forskolin

CREB, C/EBPβ and PPARγ reporter cell lines were seeded in 6-well tissue culture plates and differentiated into adipocytes as described above. Cells were treated with 10 µM of forskolin or 0.1% DMSO starting at day 0 for 48 h. TF-driven luciferase activity in the supernatants was determined and the TF activation profiles were determined as described above.

### Transcription factor network

An interaction network model of the TFs analyzed in this study was assembled based on activation relationships between the TFs documented in the published literature [11], [16], [18], [23], [24], [50], [54]–[56]. A schematic of the model is shown in Figure 4. The model included only four out of the six measured TFs, i.e. CREB, C/EBPβ, PPARγ, and SREBP-1c, as interactions involving NFAT and FoxO1 were not consistently documented in the literature. The guiding principle in assembling the model was simplicity. While it is likely that some of the TFs interact with each other through intermediaries and co-activators, these molecules were not experimentally monitored in this study, and thus it was not attempted to model these details. Including additional details could potentially improve the fit of the model to the data, but also carried the risk of over-fitting the measured data. For this reason, the activation of a TF by another factor was assumed to occur directly. The exception was the activation of PPARγ by SREBP-1c, which was assumed to involve the induction of a metabolic enzyme leading to the production of an activating ligand for PPARγ. A number of candidate ligands have been proposed such as 13-hydroxyoctadecadienoic acid, 15-hydroxyeicosatetraenoic acid and 1-O-hexadecyl-2-Azelaoyl-sn-glycero-3-phosphocholine [57], [58]. While the identity of this ligand remains to be definitively established, there is reasonable consensus that SREBP-1c activation of PPARγ involves the endogenous production of a metabolite ligand.

Including the putative PPARγ ligand, the TF network model comprises 5 species whose levels varied continuously with time. In addition, the model included three input species representing the differentiation inducing hormones insulin, dexamethasone (DEX), and isobutylmethylxanthine (IBMX). The levels of these input species were set to one (arbitrary units) or zero to reflect the addition and withdrawal of the hormones at different stages of a differentiation experiment. For the initial induction period from time zero to 48 h, the levels of all three input species were set to one. For the second induction period from 48 h to 96 h, DEX and IBMX were set to zero, while insulin was kept at one. For the maintenance period from 96 h to end of the experiment, all input species were set to zero.

The rates of change of the 5 time variant species were described using ordinary differential equations (ODEs). Partial differentiation equations or stochastic equations were not considered, because the data collected reflected the pooled averages of many (>10^6^) cells in a well-mixed environment. Two types of ODE models were created: first-order mass action kinetic models and Hill equation models. Mass action models offer the benefit of parsimony, while Hill equation models could be appropriate for networks exhibiting sigmoidal output responses to inputs [31]. By default, the ODE models implicitly assume that inputs to a node in the network are independent and additive. However, it is possible that for some TFs, multiple inputs are simultaneously required to result in activation. To explore this possibility, we utilized logical AND/OR gates to model the activation of TFs that received multiple inputs. We used an AND gate to represent the scenario where activation of a TF cannot occur unless all inputs are non-zero. Mathematically, the AND gate was represented by the minimum of all possible inputs to express the dependence of TF activation on the limiting species. We used an OR gate to model the alternative scenario where each input independently contributes to TF activation. This was mathematically represented by the sum of all inputs. The general structure of the ODEs for the mass action kinetic models was as follows.

Mass Action (OR Gate): 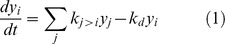


Mass Action (AND Gate): 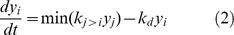


In equations (1) and (2), *y_i_* is the amount of active TF *i*, *y_j_* is the amount of a TF *j* that activates *i*, and *k_j>i_* is the rate constant for first-order activation of *i* by *j*. Every TF was assumed to decay at a rate proportional to its amount with a first-order degradation rate constant *k_d_*. The rate of production and degradation of the metabolite ligand was modeled similarly using first-order rate expressions.

The general structure of the ODEs describing the TF dynamics for the Hill equation models was as follows:

Hill Equation (OR Gate): 
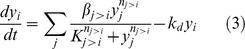


Hill Equation (AND Gate): 
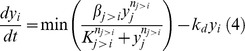


In equations (3) and (4), *y_i_, y_j_,* and *k_d_* have the same meaning as described above for the mass action kinetic models. The parameter *n_j>i_* is the Hill coefficient for the activation of *i* by *j*, which is a measure of cooperativity between substrate binding events leading to activation. The parameter *K_j>i_* is the Hill constant for the activation of *i* by *j*, and corresponds to the substrate concentration that results in half-maximal response, and β*_j>i_* is a rate constant analogous to the mass action rate constant *k_j>i_*.

Three of the TF species in the model received more than one input. These were C/EBPβ, PPARγ, and SREBP-1c. Since molecular level details needed to determine the input requirements were not available, all possible combinations of logic gates were tested. Each mass action or Hill equation model had the same respective governing equations for the CREB and ligand nodes (since these received only one input each), but included a different combination of logic gates at the C/EBPβ, PPARγ, and SREBP-1c nodes. The possible logic gates at the C/EBPβ, PPARγ, and SREBP-1c nodes are shown in Table S3. The combinations of logic gates resulting in different model structures are shown in Table S4. The 32 gate combinations shown in Table S4 were each modeled with both mass action kinetics and Hill equations, resulting in a total of 64 distinct model variants. The full set of ODEs describing mass action Model 31 and Hill equation Model 31 (all OR combinations) are shown in Tables S1 and S2, respectively.

### Parameter estimation

The model parameters (Tables S1 and S2) were estimated from the experimentally obtained TF time course data (control condition without forskolin treatment) using a nonlinear constrained optimization procedure. The objective function was to minimize the sum of squared residuals (SSR) measuring the discrepancy between the measured and calculated transcription factor levels for all time points.
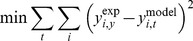
(5)


In the above expression, *y_i,t_* refers to the *i*th TF activity level (in RLU/h/RFU) at time point *t*, with the superscript denoting experimentally measured or model calculated value. The optimization variables were the model parameters. The total number of parameters in each mass action or Hill equation model was 13 or 31, respectively. The experimental data were averages of two independent experiments, where each experiment included two biological replicates for each TF. Parameter optimization was iterated 50 times. Each time, randomly generated noise drawn from a normal distribution was added to the experimental data. A noise level of 5% standard deviation was used based on average variances in the measured TF activities across replicate experiments. For each of the 50 iterations, the optimization was repeated 5 times using a randomly generated set of initial parameter values.

For the mass action models, the upper bounds on the parameters were set to 100 (day^−1^) to ensure that the first-order rate parameters are of similar magnitude as the observed rates, which were on the order of ∼10^0^ day^−1^ (due to normalization of the experimental data). The lower bounds on the TF degradation rate constants (k_2_, k_5_, k_8_ and k_10_) were set to 0.5, to ensure that there is a finite degradation rate for each of the factors. We chose a minimal rate constant value that is equivalent to a half-life of ca. 36 h, which is 5- to 10-fold longer than the reported half-lives of TF modeled in this study. The lower bounds on all other parameters were set to zero. The initial values of the TFs were set to one (‘1’) to reflect the normalization of the experimental data. The initial value of the metabolite ligand was set to zero, as it was assumed that the ligand is not present in preadipocytes prior to induction.

The bounds for the Hill equation model parameters were set identically, with the exception of the Hill coefficients (Table S2, parameters 14 through 22). These coefficients were constrained by an upper bound of 40 based on the rationale that Hill coefficient values should in principle reflect the number of total ligand binding sites on a macromolecule. The values reported in the literature are generally on the order of 10^0^ [31], [32], [59]. The parameter optimization was performed using the LSQNONLIN function (trust-region-reflective method) of the Optimization Toolbox in MATLAB (Natick, MA).

### Simulation of the transcription factor network

The TF network model described above was evaluated by comparing the simulated TF time profiles against experimental data. Model simulations were performed using all 50 sets of parameters, leading to 50 different sets of TF time profiles for each of the 64 model variants. Addition of forskolin, which rapidly increases intracellular cAMP [60], was modeled as step increase in IBMX during the first induction period (time 0 to 48 h) from a value of 1 to 1.3. This step increase in IBMX was set based on the measured profile of CREB, which is the direct target of IBMX in the model. All other model parameters and initial values were the same as the parameter estimation problem.

### Sensitivity analysis and confidence interval calculation

The sensitivity of the model simulations to the estimated parameter values was analyzed using a bootstrapping method [61]. The sensitivity analysis was performed only for the best fitting mass action model (MA31). To generate the bootstrap data, the experimental data was resampled by adding random noise drawn from a normal distribution, similar to the procedure used for parameter estimation. First, parameter optimization was performed as described above on 2,000 bootstrap datasets to generate 2,000 corresponding parameter sets, which were then used to determine the confidence intervals (CIs). The 95% CI for each parameter was found by the percentile method:
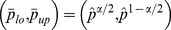
(6)


In equation (6), 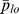 and 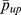 are the lower and upper confidence limits, respectively, and 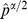 and 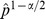 refer to the 2.5^th^ and 97.5^th^ percentiles of the dataset. Next, 2,000 *new* bootstrap data sets were generated using the resampling method. The new resampled data were then used to re-estimate the model parameters. For this re-estimation, the lower and upper bounds for the parameters were set based on the 95% CI values determined previously. The new set of parameters was then filtered for outliers as follows. For each parameter, the spread (*sp*) was defined as the interquartile range (*Q_3_–Q_1_*). Outliers were defined as parameter values that were less than *Q_1_−1.5sp* or greater than *Q_3_+1.5sp*. After removing the outliers, new CIs for the parameters were calculated from their distributions (number density functions). The length (L) and shape (sh) of the CIs were calculated as follows:
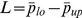
(7)




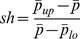
(8)


In equations (7) and (8), 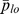 and 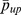 are the lower and upper parameter confidence limits, respectively, and 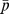 is the mean value of the parameter *p*. Larger lengths indicate less sensitivity to a given parameter, and shape values greater than 1.0 indicate nonlinearity of the model with respect to the parameters. The 95% CIs of the state variables were estimated from the TF and ligand time profiles simulated using the bootstrap parameters.

Using the bootstrap datasets that do not contain outliers as the baseline, numerical perturbation experiments were performed to quantify the change in model species (TFs and ligand) resulting from a finite change in a single parameter. Each experiment increased or decreased the value of a single parameter by a fixed fraction (e.g. 15%), while keeping all other parameters constant. The corresponding changes in the TF and ligand time profiles were calculated as follows: 
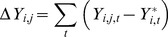
(9)


In equation (9), Y^*^_i,t_ is the activity of species *i* at time calculated using an unperturbed set of parameters, and *Y_i,j,t_* is the corresponding activity after parameter *j* has been perturbed. A one-sample *t*-test (p<0.05) was performed on the resulting sum of changes ΔY to determine whether the perturbation had a significant, non-zero effect. As every parameter significantly affected at least one model species, we also determined how far the perturbed profiles deviated from the unperturbed profiles by averaging the distribution of activity profiles for each combination of model species and parameter perturbation. The differences between the perturbed and unperturbed profiles were quantified by computing the fraction of points in the perturbed profiles that lie outside the 95% confidence intervals of the corresponding unperturbed profile.

## Supporting Information

Figure S1 3T3-L1 transcription factor reporter cells after 19 days of induction for differentiation. Transmitted light images of (A) FoxO1, (C) CREB, (E) NFAT, (G) SREBP-1c and (I) C/EBPβ and red fluorescence images of (B) FoxO1, (D) CREB, (F) NFAT, (H) SREBP-1c and (J) C/EBPβ. Scale bar  = 50 µm. **Figure S1.** 3T3-L1 transcription factor reporter cells after 19 days of induction for differentiation. Transmitted light images of (A) FoxO1, (C) CREB, (E) NFAT, (G) SREBP-1c and (I) C/EBPβ and red fluorescence images of (B) FoxO1, (D) CREB, (F) NFAT, (H) SREBP-1c and (J) C/EBPβ. Scale bar  = 50 µm.
(TIF)

Figure S2 Comparison of residuals calculated for Hill equation and mass action models. The model numbers refer to different combination of logic gates at C/EBP, PPARγ, and SREBP-1c. The specific combination of gates for each model number can be determined from Tables S3 and S4. Data shown are the best (lowest) SSR for each model variant calculated from 50 repeated optimization runs (see Materials and Methods, Parameter optimization for additional details).
(TIFF)

Figure S3 Effect of IBMX input concentration on model simulation. Data shown are plots of (A) CREB, (B) C/EBP, and (C) PPARγ activity time profiles generated using the best fitting mass action model (MA31) with different IBMX concentrations for the initial induction period. The optimal IBMX input resulting in the best fit of the model to the forskolin data was determined to be 1.4 (i.e. a 40% increase over the induction experiment without forskolin). This was determined by optimizing on the IBMX input level using the TF data from the forskolin experiment, with all other parameters set to the best fit values from the training data without forskolin. Varying the IBMX concentration from 1.0 (i.e. without forskolin added) to 1.7 had no significant impact on the profiles for C/EBP and PPARγ. The input IBMX levels used for the different activity profiles are specified in the figure legend.
(TIFF)

Figure S4 Relationship between cell number, red fluorescence intensity, and luciferase activity. (A) Different numbers of 3T3-L1 FoxO1 reporter cells were seeded in a 24-well plate and red fluorescence intensity (RFU) was measured at 550 nm (excitation) and 600 nm (emission) after 6 h. (B) A 3T3-L1 cell line with a non-specific transcription factor binding site (AGTTGGACGCGAC) was generated as described in Materials and Methods. Cells were seeded at different numbers in a 24-well plate and the luciferase activity in the supernatant determined after 24 h along with measurement of RFU in the cells.
(TIF)

Table S1 Mass action model equations and parameters. The parameter values correspond to the best fitting mass action model (MA31), which has OR gates at every node in the TF network.
(DOCX)

Table S2 Hill equation model equations and parameters. The parameter values correspond to the best fitting Hill equation model (HE31), which has OR gates at every node in the TF network.
(DOCX)

Table S3 Possible logic gate combinations at C/EBP, PPARγ, and SREBP-1c.
(DOCX)

Table S4 Model variants representing different logic gate combinations. Each model row corresponds to a specific model variant. Each model variant, two types of ODE models were implemented, mass action and Hill equation, resulting in a total of 64 model variant-ODE combinations.
(DOCX)

Dataset S1 Fold change (RLU/h/RFU) in the activation of different TF during adipocyte differentiation and maintenance. RLU/h/RFU values were determined by normalizing the RLU/h/RFU at each time point to the corresponding value at the start of differentiation (i.e., day 0). Data are from two independent experiments and represent mean ± SD. *: p<0.05.
(XLSX)

Dataset S2 Effect of parameter perturbations on calculated model species. The perturbed parameters refer to the besting fitting mass action model (MA31), with the parameters indicated by their numbers (in rows). Each block of columns describes the effect of a 15% increase or decrease on the change on a particular model species, as calculated by equation (9). The mean change in model species, standard deviation, and the CIs are shown in the corresponding columns. The *t*-test column shows the results of a one-sample *t*-test performed on the sum of changes in the model species. A value of 1 in the *t*-test column indicates that the sum of changes in the model species resulting from the parameter perturbation was significantly different from zero (*p*<0.05). See Methods for additional details.
(XLSX)

Dataset S3 Fraction of calculated model species values that lie outside of the 95% CI bounds of the unperturbed baseline case after the indicated parameter has been perturbed. The perturbed parameters refer to the besting fitting mass action model (MA31), with the parameters indicated by their numbers (in rows). Each block of columns describes the results of a different level of perturbation (ranging from +/−25 to +/−75%). Each column in the block describes the results for a different model species, as identified by the heading. For a given time point in the numerically integrated time profile, whether a perturbed species value lies outside of the CI bounds was determined based on the mean of the bootstrap values. Each calculated model species profile comprised 88 discrete, equally spaced integration time steps.
(XLSX)

Dataset S4 Fold change (RLU/h/RFU) in the activation CREB, C/EBP and PPARγ upon addition of 10 µM of forskolin or 0.1% DMSO at day 0 for 48 h. RLU/h/RFU values were determined by normalizing the RLU/h/RFU at each time point to the corresponding value at the start of differentiation (i.e., day 0). Data are from two independent experiments and represent mean ± SD. *: p<0.05.
(XLSX)
