# Supplementary figures and images for: Analysis of Transcription Factor Network Underlying 3T3-L1 Adipocyte Differentiation
Source: PLoS One. 2014 Jul 30;9(7):e100177. doi: 10.1371/journal.pone.0100177 (PMC4116336; doi:10.1371/journal.pone.0100177)

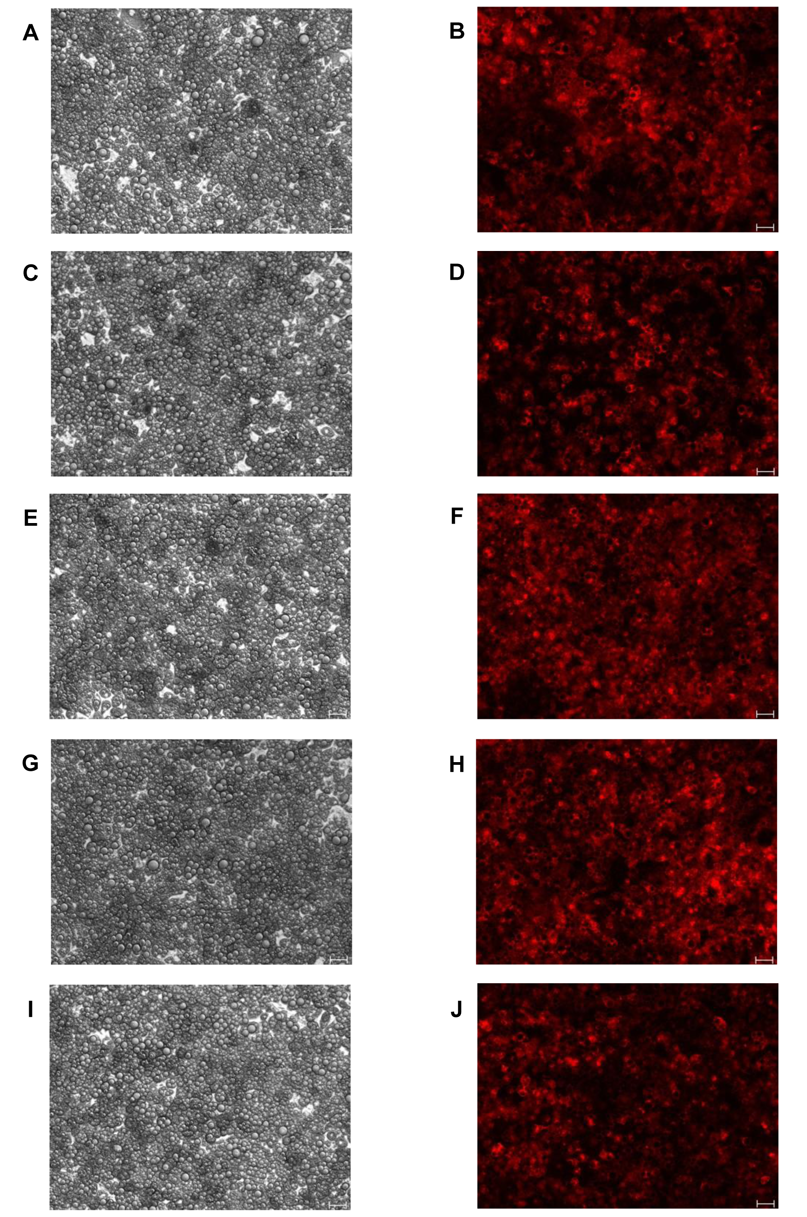

Supplement: Figure S1 — 3T3-L1 transcription factor reporter cells after 19 days of induction for differentiation. Transmitted light images of (A) FoxO1, (C) CREB, (E) NFAT, (G) SREBP-1c and (I) C/EBPβ and red fluorescence images of (B) FoxO1, (D) CREB, (F) NFAT, (H) SREBP-1c and (J) C/EBPβ. Scale bar = 50 µm. Figure S1. 3T3-L1 transcription factor reporter cells after 19 days of induction for differentiation. Transmitted light images of (A) FoxO1, (C) CREB, (E) NFAT, (G) SREBP-1c and (I) C/EBPβ and red fluorescence images of (B) FoxO1, (D) CREB, (F) NFAT, (H) SREBP-1c and (J) C/EBPβ. Scale bar = 50 µm. (TIF) [file pone.0100177.s001.tif]

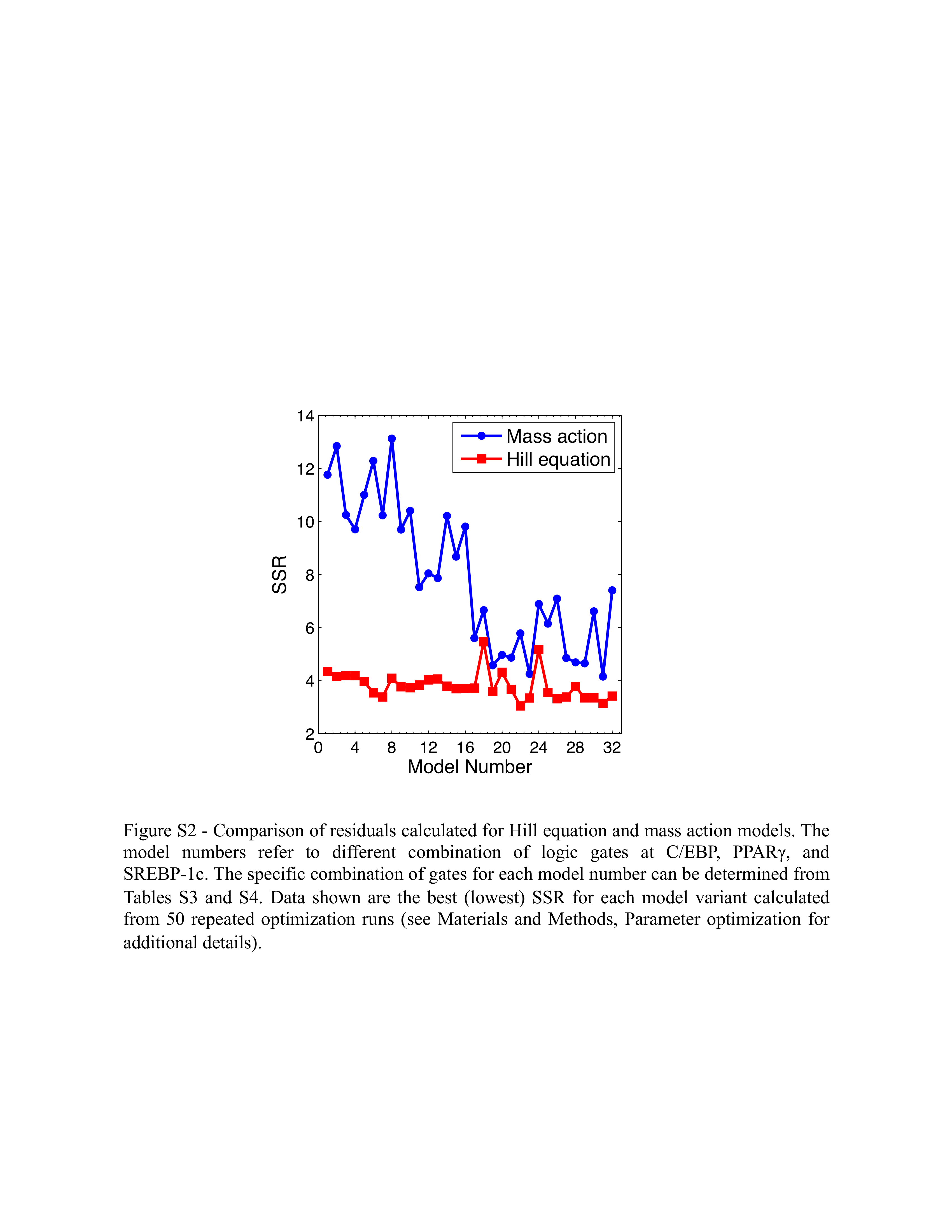

Supplement: Figure S2 — Comparison of residuals calculated for Hill equation and mass action models. The model numbers refer to different combination of logic gates at C/EBP, PPARγ, and SREBP-1c. The specific combination of gates for each model number can be determined from Tables S3 and S4. Data shown are the best (lowest) SSR for each model variant calculated from 50 repeated optimization runs (see Materials and Methods, Parameter optimization for additional details). (TIFF) [file pone.0100177.s002.tiff]

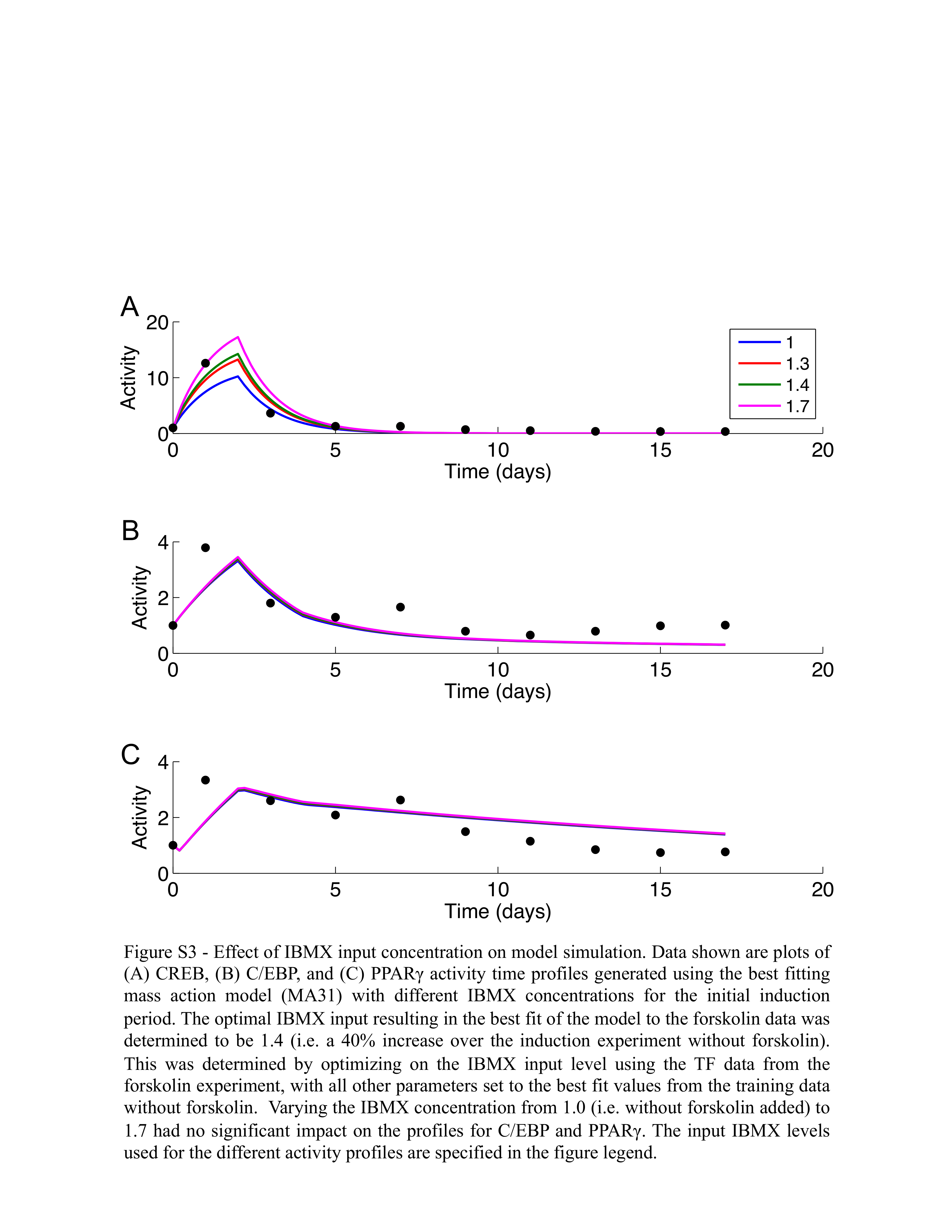

Supplement: Figure S3 — Effect of IBMX input concentration on model simulation. Data shown are plots of (A) CREB, (B) C/EBP, and (C) PPARγ activity time profiles generated using the best fitting mass action model (MA31) with different IBMX concentrations for the initial induction period. The optimal IBMX input resulting in the best fit of the model to the forskolin data was determined to be 1.4 (i.e. a 40% increase over the induction experiment without forskolin). This was determined by optimizing on the IBMX input level using the TF data from the forskolin experiment, with all other parameters set to the best fit values from the training data without forskolin. Varying the IBMX concentration from 1.0 (i.e. without forskolin added) to 1.7 had no significant impact on the profiles for C/EBP and PPARγ. The input IBMX levels used for the different activity profiles are specified in the figure legend. (TIFF) [file pone.0100177.s003.tiff]
