# Supplementary material for: Analysis of Transcription Factor Network Underlying 3T3-L1 Adipocyte Differentiation
Source: PLoS One. 2014 Jul 30;9(7):e100177. doi: 10.1371/journal.pone.0100177 (PMC4116336; doi:10.1371/journal.pone.0100177)
Supplement: Table S1 — Mass action model equations and parameters. The parameter values correspond to the best fitting mass action model (MA31), which has OR gates at every node in the TF network. (DOCX) [file pone.0100177.s005.docx]

**Table S1.** Mass action model equations and parameters. The parameter values correspond to the best fitting mass action model (MA31), which has OR gates at every node in the TF network.

| **Model Species** | **Differential equation** | ***k*** | **min** | **max** | **median** | ***k_lo_^2.5%^*** | ***k_up_^97.5%^*** | **L** | **sh** |
| --- | --- | --- | --- | --- | --- | --- | --- | --- | --- |
| CREB | **$\frac{dCREB}{dt}=k_{1}IBMX-k_{2}CREB$** | *k_1_* | 9.67 | 15.10 | 12.50 | 10.60 | 14.50 | 3.92 | 1.01 |
|  |  | *k_2_* | 0.81 | 1.16 | 0.99 | 0.86 | 1.11 | 0.25 | 1.02 |
| C/EBPβ | **$\frac{dCEBP}{dt}=k_{3}CREB+k_{4}DEX+k_{7}PPAR-k_{5}CEBP$** | *k_3_* | 0.00 | 0.10 | 0.04 | 0.00 | 0.08 | 0.08 | 1.29 |
|  |  | *k_4_* | 1.04 | 2.43 | 1.78 | 1.29 | 2.21 | 0.92 | 1.00 |
|  |  | *k_5_* | 0.50 | 0.60 | 0.50 | 0.50 | 0.53 | 0.03 | 8.62 |
|  |  | *k_7_* | 0.09 | 0.14 | 0.10 | 0.09 | 0.12 | 0.02 | 1.27 |
| PPARγ | **$\frac{dPPAR}{dt}=k_{6}CEBP+k_{12}Ligand-k_{8}PPAR$** | *k_6_* | 0.00 | 18.50 | 0.48 | 0.00 | 13.60 | 13.60 | 2.83 |
|  |  | *k_8_* | 0.50 | 27.50 | 7.22 | 0.50 | 20.60 | 20.10 | 1.96 |
|  |  | *k_12_* | 0.20 | 7.47 | 0.31 | 0.22 | 6.57 | 6.35 | 5.78 |
| SREBP-1c | **$\frac{dSREBP}{dt}=k_{9}Insulin+k_{13}PPAR-k_{10}SREBP$** | *k_9_* | 0.30 | 0.80 | 0.60 | 0.46 | 0.74 | 0.28 | 1.24 |
|  |  | *k_10_* | 0.50 | 1.78 | 0.50 | 0.50 | 1.70 | 1.20 | 3.72 |
|  |  | *k_13_* | 0.13 | 0.67 | 0.15 | 0.13 | 0.67 | 0.54 | 3.31 |
| Ligand | **$\frac{dLigand}{dt}=k_{11}SREBP-k_{12}Ligand$** | *k_11_* | 0.71 | 56.70 | 16.50 | 0.80 | 44.20 | 43.40 | 1.89 |
