# Supplementary material for: Analysis of Transcription Factor Network Underlying 3T3-L1 Adipocyte Differentiation
Source: PLoS One. 2014 Jul 30;9(7):e100177. doi: 10.1371/journal.pone.0100177 (PMC4116336; doi:10.1371/journal.pone.0100177)
Supplement: Table S2 — Hill equation model equations and parameters. The parameter values correspond to the best fitting Hill equation model (HE31), which has OR gates at every node in the TF network. (DOCX) [file pone.0100177.s006.docx]

**Table S2.** Hill equation model equations and parameters. The parameter values correspond to the best fitting Hill equation model (HE31), which has OR gates at every node in the TF network.

| **Model Species** | **Differential equation** | **P** | **Parameter** | **min** | **max** | **median** |
| --- | --- | --- | --- | --- | --- | --- |
| CREB | **$\frac{dCREB}{dt}=\frac{\beta_{1}IBMX^{n_{1}}}{K_{1}^{n_{1}}+IBMX^{n_{1}}}-k_{2}CREB$** | 1 | *K_1_* | 0.04 | 0.93 | 0.44 |
|  |  | 2 | *k_2_* | 0.43 | 1.08 | 0.90 |
|  |  | 3 | *K_3_* | 0.11 | 2.67 | 0.38 |
|  |  | 4 | *K_4_* | 0.01 | 3.51 | 0.62 |
| C/EBPβ | **$\frac{dCEBP}{dt}=\frac{\beta_{3}CREB^{n_{3}}}{K_{3}^{n_{3}}+CREB^{n_{3}}}+\frac{\beta_{4}DEX^{n_{4}}}{K_{4}^{n_{4}}+DEX^{n_{4}}}+\frac{\beta_{7}PPAR^{n_{7}}}{K_{7}^{n_{7}}+PPAR^{n_{7}}}-k_{5}CEBP$** | 5 | *k_5_* | 0.23 | 3.16 | 1.24 |
|  |  | 6 | *K_6_* | 0.00 | 14.20 | 0.40 |
|  |  | 7 | *K_7_* | 0.00 | 4.66 | 0.12 |
|  |  | 8 | *k_8_* | 0.24 | 1.53 | 0.70 |
|  |  | 9 | *K_9_* | 0.02 | 1.05 | 0.40 |
|  |  | 10 | *k_10_* | 0.29 | 2.27 | 0.99 |
|  |  | 11 | *K_11_* | 0.00 | 69.50 | 0.68 |
|  |  | 12 | *K_12_* | 0.00 | 64.80 | 0.59 |
|  |  | 13 | *K_13_* | 0.00 | 1.48 | 0.39 |
|  |  | 14 | *n_1_* | 0.56 | 7.92 | 5.17 |
| PPARγ | **$\frac{dPPAR}{dt}=\frac{\beta_{6}CEBP^{n_{6}}}{K_{6}^{n_{6}}+CEBP^{n_{6}}}+\frac{\beta_{12}Ligand^{n_{12}}}{K_{12}^{n_{12}}+Ligand^{n_{12}}}-k_{8}PPAR$** | 15 | *n_3_* | 0.52 | 21.50 | 0.94 |
|  |  | 16 | *n_4_* | 0.60 | 3.29 | 1.40 |
|  |  | 17 | *n_6_* | 0.52 | 30.70 | 7.82 |
|  |  | 18 | *n_7_* | 0.50 | 7.93 | 0.91 |
|  |  | 19 | *n_9_* | 0.52 | 2.08 | 1.17 |
|  |  | 20 | *n_11_* | 0.50 | 39.80 | 5.51 |
|  |  | 21 | *n_12_* | 0.50 | 27.00 | 1.06 |
| SREBP-1c | **$\frac{dSREBP}{dt}=\frac{\beta_{9}Insulin^{n_{9}}}{K_{6}^{n_{9}}+Insulin^{n_{9}}}+\frac{\beta_{13}PPAR^{n_{13}}}{K_{13}^{n_{13}}+PPAR^{n_{13}}}-k_{10}SREBP$** | 22 | *n_13_* | 0.50 | 25.80 | 0.89 |
|  |  | 23 | *β_1_* | 0.84 | 18.00 | 11.80 |
|  |  | 24 | *β_3_* | 0.51 | 6.11 | 2.30 |
|  |  | 25 | *β_4_* | 0.02 | 5.90 | 1.46 |
|  |  | 26 | *β_6_* | 0.00 | 4.00 | 1.59 |
|  |  | 27 | *β_7_* | 0.12 | 1.55 | 0.66 |
|  |  | 28 | *β_9_* | 0.17 | 2.61 | 1.16 |
| Ligand | **$\frac{dLigand}{dt}=\frac{\beta_{11}SREBP^{n_{11}}}{K_{11}^{n_{11}}+SREBP^{n_{11}}}-k_{12}Ligand$** | 29 | *β_11_* | 0.00 | 72.40 | 1.63 |
|  |  | 30 | *β_12_* | 0.00 | 75.50 | 1.47 |
|  |  | 31 | *β_13_* | 0.13 | 2.58 | 0.97 |
