# Supplementary material for: Analysis of Transcription Factor Network Underlying 3T3-L1 Adipocyte Differentiation
Source: PLoS One. 2014 Jul 30;9(7):e100177. doi: 10.1371/journal.pone.0100177 (PMC4116336; doi:10.1371/journal.pone.0100177)
Supplement: Table S3 — Possible logic gate combinations at C/EBP, PPARγ, and SREBP-1c. (DOCX) [file pone.0100177.s007.docx]

**Table S3.** Possible logic gate combinations at C/EBP, PPARγ, and SREBP-1c.

|  | **C/EBP** | **PPARγ** | **SREBP** |
| --- | --- | --- | --- |
| **1** | (CREB OR DEX)  AND PPARγ | C/EBP AND LIGAND | INSULIN AND PPARγ |
| **2** | (CREB OR PPARγ) AND DEX | C/EBP OR  LIGAND | INSULIN OR  PPARγ |
| **3** | (DEX OR PPARγ) AND CREB |  |  |
| **4** | (CREB AND DEX) OR PPARγ |  |  |
| **5** | (CREB AND PPARγ) OR DEX |  |  |
| **6** | (DEX AND PPARγ)  OR CREB |  |  |
| **7** | CREB OR  DEX OR PPARγ |  |  |
| **8** | CREB AND  DEX AND PPARγ |  |  |
