# Supplementary material for: Analysis of Transcription Factor Network Underlying 3T3-L1 Adipocyte Differentiation
Source: PLoS One. 2014 Jul 30;9(7):e100177. doi: 10.1371/journal.pone.0100177 (PMC4116336; doi:10.1371/journal.pone.0100177)
Supplement: Table S4 — Model variants representing different logic gate combinations. Each model row corresponds to a specific model variant. Each model variant, two types of ODE models were implemented, mass action and Hill equation, resulting in a total of 64 model variant-ODE combinations. (DOCX) [file pone.0100177.s008.docx]

**Table S4.** Model variants representing different logic gate combinations. Each model row corresponds to a specific model variant. For each model variant, two types of ODE models were implemented, mass action and Hill equation, resulting in a total of 64 model variant-ODE combinations.

| **Model No.** | **C/EBP** | **PPARγ** | **SREBP** |
| --- | --- | --- | --- |
| **1** | 1 | 1 | 1 |
| **2** | 2 | 1 | 1 |
| **3** | 3 | 1 | 1 |
| **4** | 4 | 1 | 1 |
| **5** | 5 | 1 | 1 |
| **6** | 6 | 1 | 1 |
| **7** | 7 | 1 | 1 |
| **8** | 8 | 1 | 1 |
| **9** | 1 | 2 | 1 |
| **10** | 2 | 2 | 1 |
| **11** | 3 | 2 | 1 |
| **12** | 4 | 2 | 1 |
| **13** | 5 | 2 | 1 |
| **14** | 6 | 2 | 1 |
| **15** | 7 | 2 | 1 |
| **16** | 8 | 2 | 1 |
| **17** | 1 | 1 | 2 |
| **18** | 2 | 1 | 2 |
| **19** | 3 | 1 | 2 |
| **20** | 4 | 1 | 2 |
| **21** | 5 | 1 | 2 |
| **22** | 6 | 1 | 2 |
| **23** | 7 | 1 | 2 |
| **24** | 8 | 1 | 2 |
| **25** | 1 | 2 | 2 |
| **26** | 2 | 2 | 2 |
| **27** | 3 | 2 | 2 |
| **28** | 4 | 2 | 2 |
| **29** | 5 | 2 | 2 |
| **30** | 6 | 2 | 2 |
| **31** | 7 | 2 | 2 |
| **32** | 8 | 2 | 2 |
